# Biogenic *Caralluma sinaica*-derived silver nanoparticles as a synergistic antibacterial and osteoinductive nanoplatform for osteomyelitis management

**DOI:** 10.3389/fmed.2026.1773089

**Published:** 2026-05-26

**Authors:** Munirah F. Aldayel, Enas M. Ali, Basem M. Abdallah

**Affiliations:** Department of Biological Sciences, College of Science, King Faisal University, Al-Ahsa, Saudi Arabia

**Keywords:** antibacterial, *Caralluma sinaica*, osteoinductive, osteomyelitis, silver nanoparticles

## Abstract

**Introduction:**

Osteomyelitis is an inflammatory bone disease caused by infectious microorganisms. Challenges in treating chronic osteomyelitis include antibiotic resistance, inadequate immune response, and the need for surgical debridement. As an alternative treatment strategy, we developed a formulation of green phytosynthesized silver nanoparticles (AgNPs) and plant extract, as a novel multifunctional drug to achieve antibacterial and osteogenic activities in osteomyelitis.

**Methods:**

Green silver nanoparticles (CS-AgNPs) were successfully phytosynthesized using *Caralluma sinaica* methanolic leaf extract. CS-AgNPs exhibited strong elemental purity as verified by EDX and XRD analyses. A *Staphylococcus aureus*-induced osteomyelitis mouse model was used as an *in vivo* model. Immune-histochemical analysis was used to study the effect of CS-AgNPs/PE on infected bone.

**Results:**

Functionally, CS-AgNPs, combined with plant extract (CS-AgNPs/PE), exhibited significant antibacterial potency. This hybrid formulation significantly reduced MIC and MBC values against *Staphylococcus aureus* and produced extensive membrane disruption, leakage of intracellular constituents, and higher ROS production. Additionally, CS-AgNPs/PE suppressed the development of biofilms, hemolysis production, and bacterial cell wall integrity. CS-AgNPs/PE reported no cellular toxicity *in vitro* and *in vivo*. Local treatment of IOM mice with CS-AgNPs/PE significantly inhibited bacterial burden and inflammatory response associated with reduced CD68-positive macrophage infiltration. Immune-histochemical analysis of treated infected bone area with CS-AgNPs/PE revealed significantly increased osteocalcin-positive osteoblasts, collagen deposition, osteoid thickness, and mineralized bone as compared to the non-treated group. A comparative study of different treatment groups in IOM mice attributed the osteoinductive effect of CS-AgNPs/PE to the phytochemicals of the PE.

**Conclusion:**

Our data provide a novel green nanoparticles formulation with dual anti-bacterial and osteoinductive actions as a promising therapeutic strategy for chronic osteomyelitis.

## Introduction

Osteomyelitis is a bone infection caused primarily by *S. aureus*, a Gram-positive bacterium, leading to progressive bone destruction and disability (1, 2). The virulence factors of *S. aureus* invasion could act directly to lyse host bone cells and indirectly to initiate an immune response, induce inflammation, and lead to vascular impairment and necrotic bone formation (3). Therapeutic strategies for osteomyelitis involve long-term antibiotic treatment and surgical debridement, which impose significant burdens on patients and result in high costs for the healthcare system (4, 5). Furthermore, treatment of osteomyelitis has become extremely difficult due to the widespread emergence of antimicrobial resistance (6). Thus, an effective alternative drug for osteomyelitis should provide both antimicrobial effect and osteoinductive activity.

Recently, preclinical studies demonstrated the potential of composites of metal nanoparticles with antimicrobial activity, in combination with osteoinductive materials, for the treatment of osteomyelitis (7). These include, for example, the use of mesoporous silica nanoparticle-chitosan-loaded BMP-2 (8), copper (Cu)–strontium (Sr) peroxide nanoparticles (CSp NPs) (9), CaO2-loaded 2D titanium carbide nano-sheets (10), and mannose-Zn2+/Vancomycin nanoparticles (11). However, despite their powerful and versatile properties, the synthesis processes for such nanocomposites are complicated, with high costs and toxicity risks to humans and the environment (12). Conversely, plant-based nanoparticles characterized by a simple and eco-friendly synthesis process, biocompatibility, and phytochemicals as capping agents (13). Recent studies reported the effective antimicrobial, anti-inflammatory, and angiogenesis activities of green NPs in the treatment of several pathogenic diseases, including Candidiasis (14), Invasive Pulmonary Aspergillosis (15), and diabetic wound healing (16, 17). However, no studies reported the therapeutic effect of green nanoparticles in managing osteomyelitis *in vivo*. Thus, in this study, we phytosynthesized AgNPs using the plant extract of *C. sinaica* and investigated their antibacterial and osteogenic effects alone or in combination with the plant extract *in vitro* and *in vivo* in an osteomyelitis mouse model.

## Materials and methods

### Plant collection

Fresh and healthy aerial parts of *C. sinaica* were collected in May 2024 from the Al-Ahsa–Dammam Road region, Eastern Province, Saudi Arabia (approximately 25°15′N, 49°35′E; elevation ~120–150 m above sea level). The plant material was taxonomically authenticated as *C. sinaica* (family *Apocynaceae*) by a botanist at the Cairo University Herbarium, Egypt, where a voucher specimen was deposited for future reference.

### Extract preparation

The fresh leaves of *C. sinaica* were washed, dried, and powdered. The powdered material was suspended in distilled water and extracted in a water bath maintained at 70–80 °C for 25 min (18).

### Gas chromatography mass spectroscopy investigation of *Caralluma sinaica*

Dried PE was dissolved in analytical-grade methanol and filtered through a 0.22 μm membrane to remove any particulate matter. GC–MS profiling was carried out using a capillary column under a controlled temperature gradient, with helium as the carrier gas. The mass spectra obtained were compared with the NIST library database for compounds’ identification (19).

### Phytosynthesis of CS-AgNPs

Silver nanoparticles (AgNPs) were phytosynthesized by mixing 1 mL of *Caralluma sinaica* methanolic leaf extract with 10 mL of AgNO₃ solution (10–20 mM). The reaction mixture was heated at 65 °C under continuous stirring for 15 min, then incubated at room temperature for 3 h to allow complete bioreduction of Ag^+^ ions, as indicated by a color change from yellow to reddish-brown. The resulting suspension was centrifuged at 5,000 rpm for 20 min, washed thoroughly with deionized water to remove residual impurities, and then dried at 65 °C. The obtained powder was further calcined at 250 °C for 3 h and stored for subsequent use (Supplementary Figure S1) (14).

### Optimization of different parameters for CS-AgNPs phytosynthesis

To confirm the stable phytosynthesis of CS-AgNPs, key physicochemical factors, including pH, sunlight exposure time, AgNO₃ concentration, and extract dilution, were analytically optimized. Reaction mixtures containing 2 mM AgNO₃ were adjusted to pH values of 3, 5, 7, 9, and 11 using 0.1 N HCl or 0.1 N NaOH. The effect of sunlight was assessed by exposing the mixtures for up to 50 min, with sampling every 10 min. AgNO₃ concentrations (1–4 mM) and extract dilutions (1:2, 1:4, 1:8, and undiluted) were also evaluated. UV–visible spectroscopy (200–800 nm) was used to screen for CS-AgNPs formation and identify optimal phytosynsynthesis conditions. The optimized colloidal suspension was stored at room temperature for 3 months to estimate long-term stability (20).

### Characterization of CS-AgNPs

The physicochemical features of CS-AgNPs were assessed by several analytical methods. UV–visible spectroscopy (200–800 nm) established nanoparticle phytosynthesis. The crystalline nature was analyzed by X-ray diffraction (XRD; Bruker D8 Advance) using Cu Kα radiation (*λ* = 1.5406 Å). Morphological and structural properties observed via transmission and scanning electron microscopy (TEM, JEM-2100 Plus; SEM, JSM-IT800, JEOL Ltd.), while selected area electron diffraction (SAED) established crystallinity. Functional groups accountable for nanoparticle stabilization identified by Fourier transform infrared spectroscopy (FTIR, Bruker Vertex 70) in the 4,000–400 cm^−1^ range employing the KBr pellet technique. Surface charge and stability were evaluated employing dynamic light scattering (DLS) and zeta potential measurements, and topographic features were examined by atomic force microscopy (AFM, Park XE-100).

### Bacterial strain and culture conditions

*Staphylococcus aureus* strain (ACLT 32571) used in all microbiological experiments was kindly provided through a collaborative research network from previously characterized clinical isolates. These isolates were originally obtained from patients with different clinical infections, including osteomyelitis and orthopedic-related infections, and were identified and characterized using standard microbiological and biochemical methods. The selected strain was chosen based on its clinical relevance and its representation of pathogenic isolates associated with bone infections, making it suitable for the experimental osteomyelitis model employed in this study (21), *S. aureus* was selected as the test organism due to its being the primary pathogen accountable for osteomyelitis, described by biofilm development, bone invasion, and antimicrobial resistance (3).

### *In vitro* antibacterial potential assay

The minimum inhibitory concentration (MIC) was determined by the broth microdilution method. Briefly, *S. aureus* (10^5^ CFU/mL) was cultured overnight in TSB media. Then, in a 96-well plate, 100 μL of each treatment (PE, CS-AgNPs, vancomycin, and CS-AgNPs/PE combination) at serial concentrations (8–512 μg/mL) was mixed with 100 μL of *S. aureus* suspension. After 24 h of incubation, 20 μL of resazurin (1 mg/mL) was added to each well, and the mixture was incubated for 3 h in the dark. MIC is considered the lowest concentration that stops the color change from purple to orange. For the minimal bactericidal concentration (MBC), wells showing no visible growth were sub-cultured on TSA plates and incubated for 24 h. MBC is considered the lowest concentration at which no bacterial colonies are detected. Growth curves were assessed by inoculating bacteria (10^5^ CFU/mL) in 96-well plates with different treatments at concentrations from 8 to 64 μg/mL. Absorbance at 600 nm was documented every 4 h over a 24 h period using a microplate reader (22).

### Live/dead fluorescent staining assay

1 mL of *S. aureus*-induced osteomyelitis exposed to vancomycin, PE, CS-AgNPs, or CS-AgNPs/PE groups, stained with 1 μL propidium iodide (PI) and SYTO9 dye in a dark place for 25 min. The growth of *S. aureus* was assessed employing confocal laser scanning microscopy (CLCSM, Leica TCS SP8).

### Membrane integrity and LDH leakage tests

#### Electrical conductivity

To assess variations in membrane permeability, *S. aureus* cultures were treated with each treatment for 0, 1, 2, 4, and 6 h. Cultures centrifuged at 5000 rpm for 5 min; the supernatants were diluted 10-fold in 10% glucose solution. Electrical conductivity was documented with a conductivity meter.

#### LDH measurement

Bacterial cultures at logarithmic phase (OD₆₀₀ ≈ 0.6) were exposed to the MIC of each treatment. Samples collected after 1, 2, 4, and 8 h, centrifuged at 5000 rpm for 5 min, and the cell pellets were suspended in sterile PBS. The cells were disturbed by ultra-sonication, and leaked lactate dehydrogenase (LDH) activity in the lysates was measured using a commercial LDH assay kit (Jiancheng Bioengineering, Nanjing, China) (23).

#### Intracellular constituents’ leakage

To assess membrane damage, *S. aureus* (10^6^ CFU/mL) was incubated under shaking conditions at 37 °C for 4 h with control, PE, CS-AgNPs, or their combination (at MIC). Subsequently, samples were centrifuged for 15 min, and supernatants were collected for examination of reducing sugars and proteins released from bacterial cells (24).

#### Reactive oxygen species (ROS) examination

Bacterial suspension (10^6^CFU/mL) mixed with HBSS and treated with each agent (control, PE, CS-AgNPs, combination) at their MIC values for 2 h at 37 °C. After incubation, 500 μL of NBT (1 mg/mL) was added, and the samples were incubated at 37 °C for 15 min. Then, 100 μL of 0.1 M HCl was added, and the samples were centrifuged at 1000 × *g* for 10 min. The resulting pellet was suspended in HBSS, and the absorbance was measured at 575 nm to quantify ROS generation (23).

### Morphological and intracellular alterations in *Staphylococcus aureus* cells after exposure to CS-AgNPs/PE

#### Scanning electron microscopy (SEM)

Overnight *S. aureus* cultures were treated for 6 h at 37 °C with PE, CS-AgNPs, or CS-AgNPs/PE combination at their particular MICs. Cells collected via centrifugation at 5000 × *g* for 10 min, washed with PBS, and fixed in 2.5% glutaraldehyde. Then, samples were post-fixed with 1% osmium tetroxide for 2 h. Samples were then dehydrated using a graded ethanol series and gold-coated. The morphology observed under high-resolution SEM (FEI Quanta FEG 200).

#### Transmission electron microscopy (TEM)

The bacteria were fixed with 2.5% glutaraldehyde overnight. Subsequent PBS washes, samples post-fixed with 1% OsO. After dehydration through graded ethanol, samples were immersed in a 1:1 acetone/embedding medium, then left in the embedding solution overnight. Ultrathin sections were stained and then examined using a Hitachi H-7650 transmission electron microscope to detect ultrastructural modifications.

### Synergistic inhibition of CS-AgNPs with plant extract

#### Checkerboard assay

The antibacterial interaction between CS-AgNPs and plant extract was evaluated using a standard checkerboard microdilution method. Twofold serial dilutions of the plant extract were prepared in Mueller–Hinton broth (MHB) along the horizontal axis of a 96-well microtiter plate, whereas CS-AgNPs were serially diluted and distributed along the vertical axis, generating all possible concentration combinations based on previously determined MIC values.

Each well was inoculated with 20 μL of standardized bacterial suspension, except for the negative control (MHB only). A positive control containing bacteria in MHB was included. Plates were incubated at 37 °C for 18–24 h, and bacterial growth was assessed by measuring optical density at 600 nm.

MICs of each agent alone and in combination were determined, and the interaction was expressed as the fractional inhibitory concentration index (FICI):

where:

$$\text{FICI}={\mathrm{FIC}}_{\mathrm{NPs}}+{\mathrm{FIC}}_{\mathrm{PE}}$$

$${\mathrm{FIC}}_{\mathrm{NPs}}=\frac{\mathrm{MIC}\;\text{of}\;\mathrm{CS}‐\text{AgNPs in combination}}{\mathrm{MIC}\;\text{of}\;\mathrm{CS}‐\text{AgNPs in combination}}$$

$${\mathrm{FIC}}_{\mathrm{PE}}=\frac{\mathrm{MIC}\;\text{of}\;\mathrm{PE}\;\text{in combination}}{\mathrm{MIC}\;\text{of}\;\mathrm{PE}\;\text{in combination}}$$

Interactions were interpreted as synergistic (≤0.5), additive (>0.5–1), indifferent (>1–4), or antagonistic (>4). All experiments were performed in triplicate on three independent occasions.

### Assessment of anti-biofilm action of CS-AgNPs/PE against *Staphylococcus aureus*-induced osteomyelitis

See Supplementary materials and methods.

#### Sample size and data analysis

No formal *a priori* power analysis was performed. The sample size (*n* = 6 animals per group) was chosen based on established practice in comparable murine osteomyelitis models and ethical considerations. Biological replicates refer to individual animals, while histomorphometric analysis (*n* = 3) represents technical replicates from three representative tissue sections per group. This distinction has been clarified in the revised manuscript and figure legends to ensure transparency and consistency.

#### Cell culture of primary mBMSCs

Bone marrow-derived mesenchymal stem cells (mBMSCs) were isolated from the femur bone of mice as described (25). mBMSCs culture in RPMI-1640 medium (Merck) supplemented with 10% fetal bovine serum (FCS) (Thermo Fisher Scientific GmbH) and 1% penicillin/streptomycin (P/S).

#### MTT cell viability assay

The effect of CS-AgNPs, PE, and their combination on BMSCs cell viability was measured using the MTT cell proliferation assay kit (Sigma-Aldrich) according to the manufacturer’s instructions.

#### Osteoblast differentiation

mBMSCs treated with osteogenic solution containing *α*-minimum essential medium (α-MEM; Thermo Fisher Scientific GmbH) supplemented with 50 mg/mL of vitamin C (Sigma-Aldrich), 10 mM β-glycerol-phosphate, 10% FBS, and 1% penicillin/streptomycin (P/S). Cells were induced with osteogenic medium for 1 week.

#### ALP activity assay

Cells were induced to differentiate into osteoblasts in the absence or presence of various treatments for 7 days. ALP activity was measured as described (25). The CellTiter-Blue® cell viability assay was used to determine cell viability according to the manufacturer’s instructions. The value of ALP activity normalization to the cell viability is presented as a fold change over control cells.

#### Alkaline phosphatase staining

Cells fixed with acetone/citrate buffer pH 4.2 and stained with Napthol-AS-TR-phosphate solution (Merck) for 1 h at room temperature, as described (26).

#### Alizarin red S staining and quantification

BMSCs were induced to osteoblast differentiation for 7 days, fixed with 70% ice-cold ethanol at −20 °C, and stained with Alizarin red (ALZ) for 10 min at RT. For ALZ quantification, dye was eluted with 10% cetylpyridinium chloride for 1 h at RT. The absorbance was measured at 570 nm, and the data were presented as fold change over control after normalization to cell number.

#### Establishment of *Staphylococcus aureus*-induced osteomyelitis mouse model (IOM)

The IOM mouse model was approved by the Research Ethics Committee of the Deanship of Scientific Research at King Faisal University (KFU-REC-2025-NOV-ETHICS3747). Male C57BL/6 mice (2 months old, 25 ± 5 g) were used to establish the osteomyelitis model as described previously (27). Briefly, mice were anesthetized with isoflurane inhalation, and a longitudinal skin incision was made to expose the proximal tibia. A hole (1.2 mm in diameter) was drilled into the tibia metaphysis using a sterile dental bur under constant saline irrigation. A suspension of *S. aureus* (5.4 × 10^7^ CFU/mL) was inoculated into the tibia cavity to induce osteomyelitis. The wound was sutured, and the animals were monitored during recovery. Three weeks after successful establishment of the IOM mouse model, the infected site was re-exposed, and the osteomyelitic cavity was thoroughly irrigated with sterile PBS. The animals were randomly divided into six groups (*n* = 6) as follows. Group 1: sham non-infected control; Group 2: *S. aureus*-infected, non-treated control; Group 3: infected and PE-treated group; Group 4: infected and CS-AgNPs-treated group; Group 5: infected and CS-AgNPs/PE-treated group; Group 6: infected and vancomycin-treated positive control. Treatments applied locally into the tibia defect site immediately following the establishment of infection with a 20-μL aliquot of 10% ethanol in propylene glycol (vehicle), and 50 μg/mL of either PE, CS-AgNPs, or CS-AgNPs/PE 10–20 μL per tibia. Vancomycin was delivered locally as 10 μL of a 10 mg/mL solution. Treatments were repeated every 48 h for a total duration of 14 days. At the endpoint, tibiae were harvested for bacterial viability assays, histological evaluations, CD68 immunostaining, OCN immunohistochemistry, and Masson’s and Goldner’s trichrome staining.

#### Bacterial viability assessment

At the endpoint, tibiae were aseptically harvested and homogenized in sterile PBS. Serial dilutions were plated on nutrient agar to enumerate viable bacterial colonies.

#### Immunohistochemistry

Tibiae decalcified in 10% EDTA, paraffin-embedded, and sectioned at 5 μm. Sections were deparaffinized and rehydrated before incubation with rat anti-mouse CD68 antibody (Bio-Rad) or anti-OCN antibody (AB93876) (Abcam). After secondary antibody incubation and DAB chromogen development, slides were counterstained with hematoxylin. Positive cells were semi-quantitatively assessed using Image-Pro Plus 6.0 image analysis software.

For Masson’s trichrome staining. Nuclei were first stained with Weigert’s iron hematoxylin. Biebrich Scarlet-Acid Fuchsin solution (Merck) was applied to cytoplasm and unmineralized osteoid, followed by differentiation in phosphomolybdic/phosphotungstic acid. Collagen fibers counterstained with aniline blue. For assessment of bone mineralization, bone sections were subjected to Goldner’s trichrome staining. After standard deparaffinization, nuclei were stained with Weigert’s hematoxylin. Acid fuchsin-Orange G stained osteoid, while Light Green SF yellowish (Merck) labeled mineralized bone matrix. Histomorphometric analyses for mineralized bone area and osteoid thickness were performed using Image-Pro Plus 6.0 image analysis software (27).

### Statistical analysis

Data are expressed as mean ± SD. Statistical analysis was performed using one-way analysis of variance (ANOVA) followed by Tukey’s *post-hoc* test for multiple group comparisons. For comparisons between two groups, the unpaired Student’s *t*-test was used. A *p*-value <0.05 was considered statistically significant.

## Results

### Phytochemical screening of *Caralluma sinaica* leaf extract

The chemical profile of *C. sinaica* extended across multiple classes of biomolecules, comprising esters, steroids, terpenes, aliphatic alcohols, fatty acids, and flavonoids, reflecting the structural complexity and pharmacological promise of *C. sinaica* (Table 1). The GC–MS profiling of *C. sinaica* methanolic leaf extract showed a different spectrum of phytochemical components (Supplementary Figure S1, Table 2). Amongst the identified compounds, oleic acid was the major component, accounting for about 39.75% of the total composition. Other important fatty acids, namely n-hexadecanoic acid (20.88%) and octadecanoic acid (11.28%), contributed expressively to the lipid fraction.

**Table 1:** Preliminary phytochemical analysis of methanolic *Caralluma sinaica* extract.

No.	Secondary metabolites	Extract
1	Tannins	−
2	Saponins	++
3	Flavonoids	++
4	Alkaloids	++
5	phenols	+
6	Steroids	+
7	Terpenoids	+
8	Carbohydrates	+
9	Phlobatannins	−

**Table 2:** GC–MS profile of *Caralluma sinaica* methanolic extract.

No.	Compound name	Retention time (min)	Peak area (%)
1	4H-Pyran-4-one, 2,3-dihydro-3,5-dihydroxy-6-methyl	8.52	1.07
2	5-hydroxymethylfurfural	9.76	8.53
3	2-isopropoxyethyl propionate	13.09	4.73
4	1,3 dioxane, 4,4-dimethyl	3.65	3.67
5	Humulenol-II	5.82	1.31
6	Tetradecanoic acid	16.37	2.09
7	Cyclohexanol, 3-ethenyl-3-methyl	17.09	0.84
8	Palmitoleic acid	18.23	0.97
9	n-hexadecanoic acid	18.51	20.88
10	Oleic acid	20.20	39.75
11	Octadecanoic acid	20.39	11.28
12	Dehydroabietic acid	22.80	1.33

### Phytosynthesis and optimization of CS-AgNPs

Upon incubation of the *C. sinaica* leaf extract with AgNO_3_ for 2 h, the solution progressively changed from a pale yellow to a brownish hue, an alteration due to surface plasmon resonance (SPR) (Supplementary Figure S2). CS-AgNPs displayed a strong absorption peak at 420 nm (Supplementary Figure S3A). CS-AgNPs were assessed under different physicochemical conditions to detect the optimal factors for AgNPs development and stability. Changing the pH of the reaction mixture (3.0–11.0) showed that alkaline conditions supported effective biosynthesis. At pH 9.0, a distinctive surface plasmon resonance (SPR) band was observed at 420 nm, accompanied by a color change from light to dark brown, demonstrating rapid AgNPs formation. However, acidic pH values (3.0 and 5.0) failed to produce a characteristic SPR signal within the 400–500 nm range. Therefore, pH 9.0 is considered the optimum condition for CS-AgNPs phytosynthesis (Supplementary Figure S3B). The effect of sunlight exposure time (10–50 min) on CS-AgNPs phytosynthesis was evaluated. Progressive rises in absorbance suggested continuous CS-AgNPs production up to 40 min, after which point the reaction reached completion with a sharp SPR peak at 420 nm. Continued exposure beyond this time (50 min) led to instability and aggregation of the CS-AgNPs (Supplementary Figure S3C). Silver nitrate concentration optimized by applying 1–4 mM AgNO₃. Maximum phytosynthesis was observed at 2 mM, resulting in a stable and well-defined SPR peak. Greater concentrations (3 and 4 mM) led to red-shifted, broadened peaks and visible aggregation, signifying polydispersity and decreased stability (Supplementary Figure S3D). The effect of extract concentration was assessed using different dilutions of *C. sinaica* extract (1:2, 1:4, and 1:8, v/v). The 1:4 dilution produced the most effective CS-AgNPs phytosynthesis, resulting in a sharper SPR peak and greater CS-AgNPs yield compared to undiluted extract (Supplementary Figure S3E). The stability of CS-AgNPs under optimized conditions (pH 9.0, 2 mM AgNO₃, 1:4 extract dilution, 40 min sunlight exposure) was monitored for 6 months. No significant change in the SPR band was identified during this period, suggesting the long-term stability of the phytosynthesized CS-AgNPs (Supplementary Figure S3F).

### Characterization of CS-AgNPs

**Figure 1:**
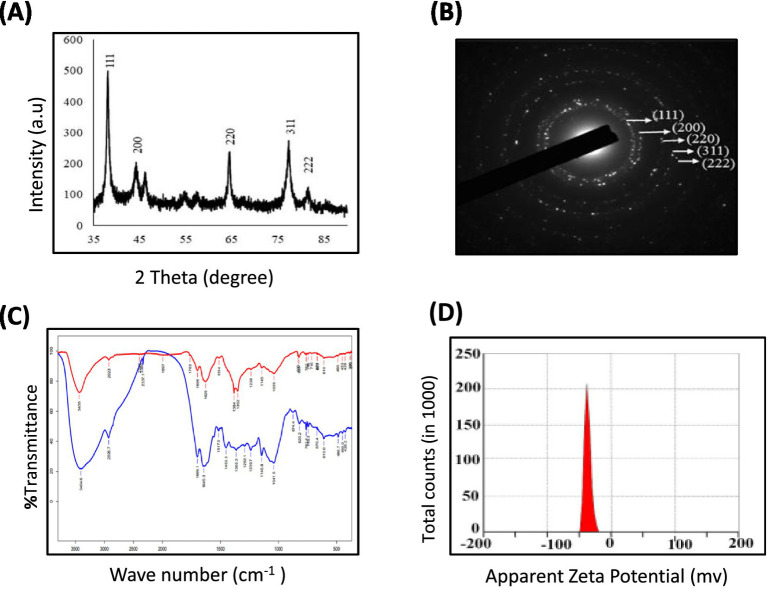
Confirmation of phytosynthesized CS-AgNPs. **(A)** XRD pattern and **(B)** SAED pattern of CS-AgNPs. **(C)** FTIR spectra for methanolic *C. sinaica* extract (blue line) and CS-AgNPs (red line). **(D)** Zeta potential is −36 mV, which is within the range for higher stability.

The crystalline nature of the CS-AgNPs was studied using X-ray diffraction (XRD). The diffraction profile showed distinctive Bragg reflections at approximately 38.2°, 44.4°, 64.5°, and 77.6°, which correspond to the (111), (200), (220), and (311) lattice planes of face-centered cubic (fcc) metallic silver, respectively. The absence of extraneous peaks confirmed the purity of the silver phase with no obvious secondary phases or impurities. The average crystallite size, calculated using the Scherrer equation, indicated that the CS-AgNPs own nanoscale crystalline domains with a constant distribution (Figure 1A). The SAED pictures showed well-defined concentric diffraction rings indexed to the (111), (200), (220), and (311) planes, confirming the polycrystalline nature of the CS-AgNPs. The presence of several diffraction rings indicates the aggregation of multiple crystalline grains within a single nanoparticle, reflecting a stable and ordered lattice arrangement (Figure 1B).

The FTIR spectrum of the methanolic *C. sinaica* extract showed numerous absorption peaks that underwent distinctive shifts after the phytosynthesis of CS-AgNPs, indicating the contribution of phytochemicals in CS-AgNPs reduction and stabilization. A broad band at approximately 3,405 cm^−1^, assigned to O–H stretching vibrations of phenolic and alcoholic groups, slightly shifted to ~3,435 cm^−1^ in CS-AgNPs, demonstrating the participation of hydroxyl groups in capping interactions. The aliphatic C–H stretching band near 2,927 cm^−1^ persevered at ~2,923 cm^−1^, signifying retention of hydrocarbon moieties on the CS-AgNPs surface. A distinctive peak at ~2,360 cm^−1^ appeared only after CS-AgNPs phytosynthesis, consistent with carboxyl (–COOH) stretching. The strong carbonyl/C–N stretching vibration at ~1,645 cm^−1^ in the extract was detected at ~1,629 cm^−1^ in CS-AgNPs, authorizing the coordination of carbonyl and amide groups with silver. A novel band at ~1,384 cm^−1^ appeared after phytosynthesis and was attributed to phenolic groups. The C–O stretching peak around 1,042 cm^−1^ shifted slightly to ~1,040 cm^−1^, consistent with stabilization by alcohols or ethers. Remarkably, a small band at ~386 cm^−1^ appeared in the CS-AgNPs spectrum, attributed to Ag–O vibrations and verifying the successful phytosynthesis of metallic CS-AgNPs (Figure 1C). Our results showed that CS-AgNPs exhibited a zeta potential of −36 mV (Figure 1D).

**Figure 2:**
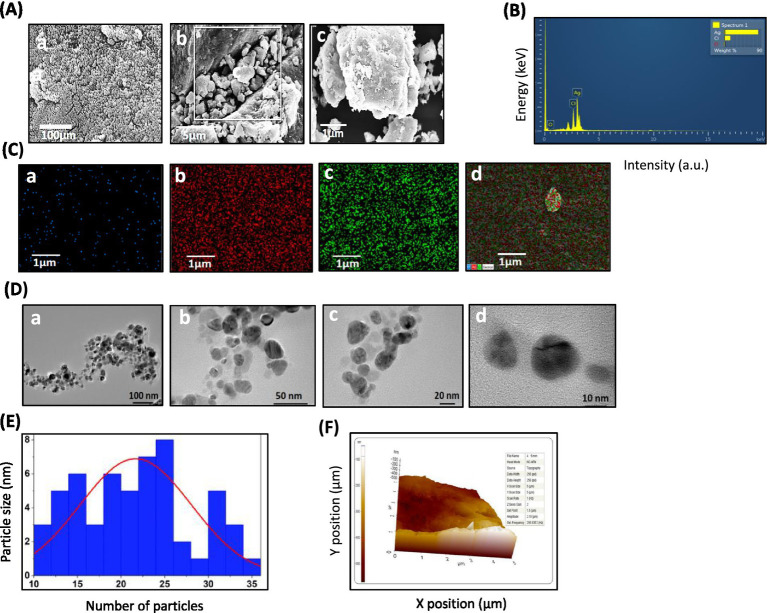
Characterization of CS-AgNPs. **(A)** SEM images of CS-AgNPs **(a–c)** and EDS analysis of the area in the white box in **(B)**. **(C)** Elemental mapping of oxygen **(a)**, silver **(b)**, chlorine **(c)**, and all three elements **(d)**. **(D)** TEM micrograph displaying the size of CS-AgNPs **(a–d)**. **(E)** Histogram of the TEM image, and **(F)** AFM graph of CS-AgNPs.

SEM micrographs of CS-AgNPs revealed a spherical shape, with a tendency to form aggregates due to their high surface energy and nanoscale dimensions. The particles appeared well-defined and had a fairly uniform distribution (Figure 2A, a–c). The EDX spectrum (Figure 2B) showed a strong, sharp peak at approximately 3 keV, consistent with silver (Ag), which is a distinctive absorption signal for metallic silver. Insignificant signals attributed to oxygen and chlorine were also identified, possibly due to the phytochemicals of the plant extract. Elemental mapping provides additional evidence for the homogeneous distribution of silver through the sample. The red and green overlays matching silver and chloride, respectively, confirmed uniform spatial localization (Figures 2C, a–c). The combined mapping (Figures 2C, d) emphasized the co-localization of elements, confirming the successful phytosynthesis of stable CS-AgNPs without phase segregation.

The TEM images confirmed that CS-AgNPs were mostly spherical, with smooth edges and well-defined boundaries (Figures 2D, a–d). The particle size distribution histogram (Figure 2E) showed that most CS-AgNPs were approximately 10–35 nm, with an average particle size of about 22–25 nm. The AFM micrographs (Figure 2F) showed a uniform dispersion of mostly spherical nanoparticles with no evidence of agglomeration, authorizing the stability of the colloidal system. The height profile and two-dimensional mapping confirmed that the particles were consistently below 100 nm in diameter.

### *In vitro* antibacterial activity

**Figure 3:**
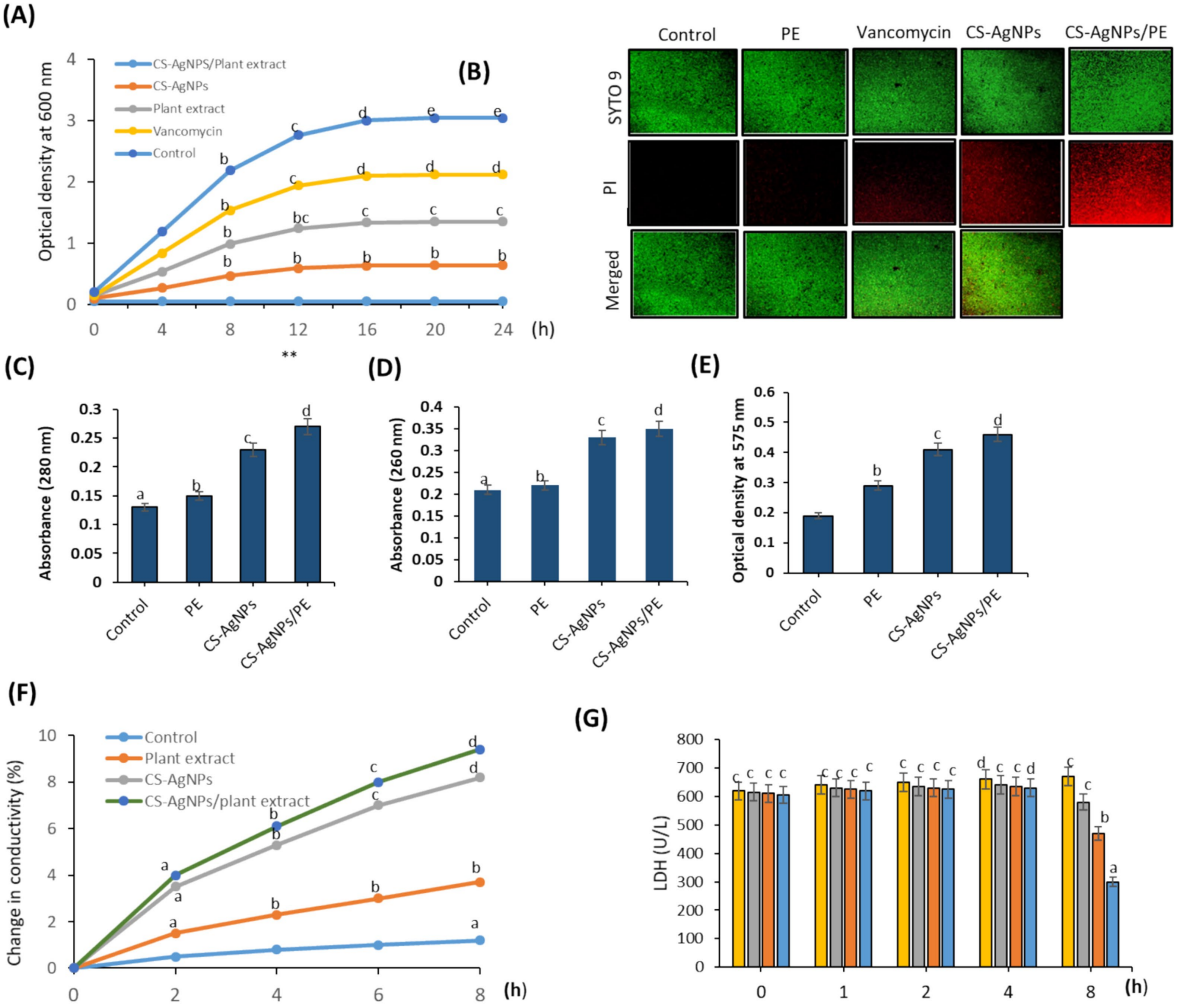
**(A)** The bactericidal activity of CS-AgNPs, PE, vancomycin, and CS-AgNPs/PE against *S. aureus*. **(B)** Confocal pictures of *S. aureus* stained by SYTO9 and PI after the treatment with CS-AgNPs, PE, vancomycin, and CS-AgNPs/PE. **(C)** Leakage of intracellular proteins from the plant extract, CS-AgNPs, and CS-AgNPs/PE-treated *S. aureus*. **(D)** Leakage of intracellular nucleic acids from the plant extract, CS-AgNPs, and CS-AgNPs/PE-treated *S. aureus*. **(E)** The intracellular ROS levels of the plant extract, CS-AgNPs, and CS-AgNPs/PE-treated *S. aureus*. **(F)** Effect of plant extract, CS-AgNPs, and CS-AgNPs/PE on the conductivity of *S. aureus*. **(G)** Effect of plant extract, CS-AgNPs, and CS-AgNPs/PE on the LDH level of *S. aureus*. Values are expressed as mean ± SD. Different letters indicate statistically significant differences (*p* < 0.05) using one-way ANOVA with Tukey’s *post hoc* test. Effect of CS-AgNPs/PE on *S. aureus* ultrastructure modification.

The MIC and MBC results confirmed that the crude plant extract showed moderate antibacterial activity. CS-AgNPs showed enhanced potential, with an MIC of 32 μg/mL and MBC of 64 μg/mL. However, the combination of CS-AgNPs with PE exhibited the most potent action, attaining the lowest MIC and MBC values of 16 μg/mL and 32 μg/mL, respectively (Table 3). The growth curve investigation supported these findings, revealing distinctive inhibitory profiles between the treatments. PE and CS-AgNPs further delayed the onset of logarithmic growth, with the combination treatment prompting the most substantial growth inhibition, efficiently stopping the formation of a typical exponential growth phase (Figure 3A). Additionally, CS-AgNPs/PE prompted the strongest antibacterial action, signifying by an intense decrease in green fluorescence and main red fluorescence signals, consistent with broad bacterial membrane compromise as assessed by fluorescence microscopy (Figure 3B).

**Table 3:** The MIC and MBC values of different treatments against *Staphylococcus aureus*-induced osteomyelitis.

Treatment	*Staphylococcus aureus*	
	MIC (μg/mL)	MBC (μg/mL)
Plant extract	64	128
CS-AgNPs	32	64
CS-AgNPs/plant extract	16	32
Vancomycin	64	128

### Effect of CS-AgNPs/PE on bacterial intracellular constituents’ leakage

Treatment of *S. aureus* with the CS-AgNPs/PE induced an obvious increase in extracellular protein and nucleic acid levels, signifying partial membrane injury. Remarkably, the combined treatment elicited the most prominent release response reflecting a strong synergistic effect (Figures 3C,D).

### Effect of CS-AgNPs/PE on bacterial ROS production

Treatment of *S. aureus* with CS-AgNPs/PE displayed a higher (59%) intracellular ROS release than PE (35%) and CS-AgNPs (48%) treatments as compared to control (Figure 3E).

### Membrane conductivity

The impact of different treatments on the membrane conductivity of *S. aureus* is shown in Figure 3F. CS-AgNPs/PE treatment displayed the highest conductivity, rising sharply to around 9–10% at the end of incubation.

### LDH release

CS-AgNPs/PE treatment induced the maximum LDH release, with values exceeding ~600 U/L. This prominent leakage highlights the synergistic membrane-disruptive action of the combined therapy (Figure 3G).

**Figure 4:**
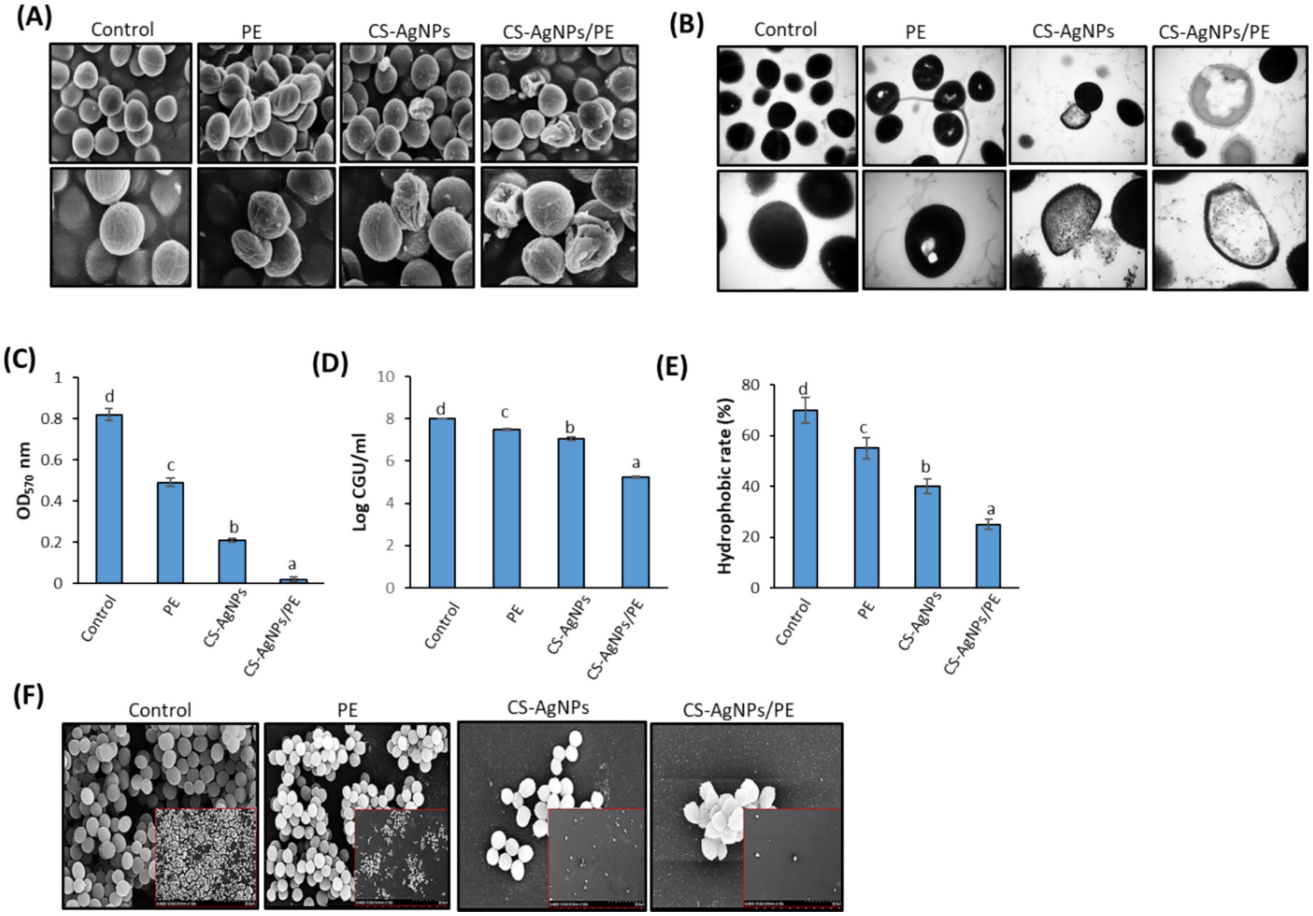
Effect of CA-AgNPs on *S. aureus* ultrastructure. **(A)** SEM images of *S. aureus* untreated or treated with different conditions. Untreated *S. aureus* cells were sphere-shaped with a complete cell wall and membrane. The shape of the plant extract-treated cells was modified and distorted. Some CS-AgNPs-treated cells lose their integrity, triggering leakage of cellular components. In combination treatment, more cells lost their regular structure, which displayed severe damage, including degeneration and cell wall injury. **(B)** TEM images of *S. aureus*, untreated or treated with different conditions. Untreated cells displayed homogeneous electron density in the cytoplasm. Plant extract-treated cells showed rough cell walls and blurred cell membranes. CS-AgNPs-treated cells exhibited apparent cytoplasmic release, cell wall, and membrane damage. In combination treatment of CS-AgNPs/PE, *S. aureus* cells were nearly in a ghost state. **(C)** The effect of different treatments on biofilm metabolic activity of *S. aureus* by MTT staining test. **(D)** Cell quantification of biofilms after different treatments. **(E)** The effects of various treatments on the hydrophobicity of *S. aureus* biofilm. **(F)** SEM images reveal the morphological changes and structural integrity of *S. aureus* biofilms following various treatments. Control biofilms exhibit dense, well-packed bacterial clusters. Treatment with plant extract displays incomplete disturbance of the biofilm. CS-AgNPs prompt marked disturbance, resulting in clear gaps between bacterial cells. The combination treatment causes nearly complete degeneration of the biofilm structure. Scale bar = 5 μm. Red boxes (10,000×) and the large images (1,500×) are magnified. Values are expressed as mean ± SD. Different letters indicate statistically significant differences (*p* < 0.05) using one-way ANOVA with Tukey’s *post hoc* test.

*Staphylococcus aureus* treated with PE alone displayed mild distortion with wrinkled cell envelope, minor membrane undulations, and heterogeneity of cytoplasmic density. However, CS-AgNPs treatment revealed more severe damage, with visible cracks appearing in the cell wall and membrane, cytoplasmic leakage, and cells appeared shrunken. Additionally, there was partial loss of membrane integrity and some collapse of internal structure. The most severe actions occurred in CS-AgNPs/PE treatment. Several cells lost apparent morphology, displaying membrane degeneration, cell wall destruction, extensive loss of cytoplasm, and abundant cell debris in the background (Figure 4A).

TEM images showed progressive ultrastructural distraction in *S. aureus* cells following different treatments. PE treatment prompted partial cytoplasmic disorganization and slight detachment of the membrane from the cell wall. CS-AgNPs resulted in more severe modifications, comprising disrupted membranes, vacuolation, and release of cytoplasmic contents. However, the combination treatment led to extensive structural collapse, with complete membrane rupture, broad cytoplasmic loss, and empty cells surrounded by debris signifying the most effective synergistic destruction (Figure 4B).

### Anti-biofilm activity of CS-AgNPs/PE against *Staphylococcus aureus*

The effect of different treatments on biofilm biomass quantified by assessing OD_570_ values (Figure 4C). CS-AgNPs/PE inhibited biofilm establishment, with negligible biomass detected (OD_570_ ≈ 0.05) and reducing bacterial counts to nearly 3 log CFU/ml compared with CS-AgNPs or PE alone (Figure 4D). Furthermore, CS-AgNPs/PE reduced the hydrophobic rate to below 20% (Figure 4E). These findings confirmed by SEM examination (Figure 4F). The treatment with the PE or CS-AgNPs alone led to obviously thinner and less compact biofilm layers. However, CS-AgNPs/PE showed the most prominent disruption, with severely impaired biofilm architecture, scarce bacterial clusters, and large areas lacking extracellular polymeric substances (Figure 4F).

### Quantitative analysis of biofilm

**Figure 5:**
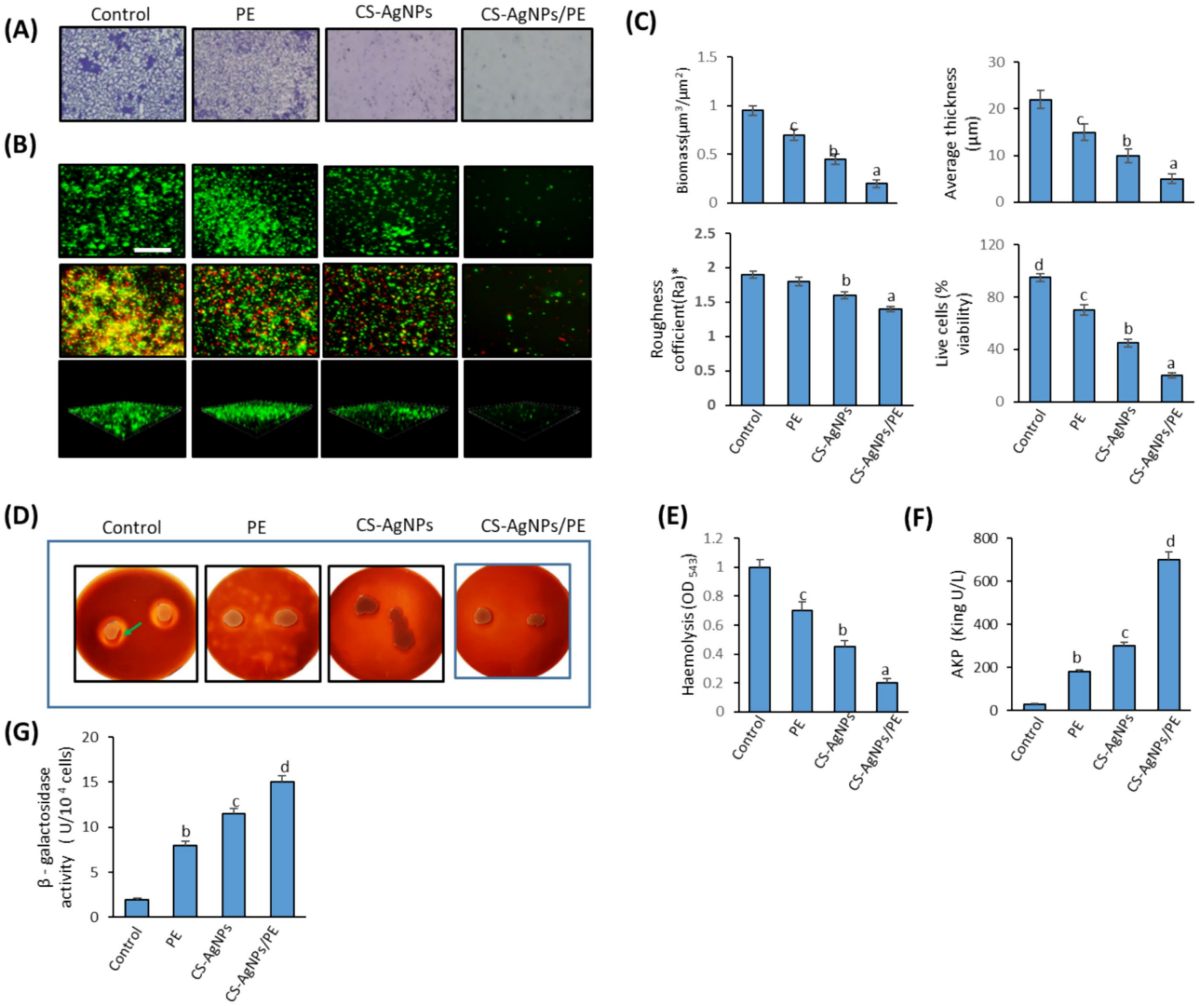
Evaluation of the anti-biofilm efficacy of plant extract, CA-AgNPs, or CA-AgNPs/PE combination against *S. aureus*. **(A)** Light microscope images of *S. aureus* biofilm after different treatments (40×). **(B)** Confocal laser scanning microscopy (CLSM) pictures demonstrating the spatial distribution and viability of *S. aureus* biofilms after different treatments. Untreated controls showed dense, thick biofilms with mostly viable cells. Treatments progressively decreased biofilm density and thickness, with the combined CS-AgNPs/PE group showing the most distinct disturbance and significantly reduced cell viability. Scale bars represent 50 μm. **(C)** Quantitative analysis of *S. aureus* biofilm architecture following different treatments. **(D)** Assessment of hemolytic activity of *S. aureus* following treatment with plant extract, CA-AgNPs, or CA-AgNPs/PE combination. **(E)** Quantitative evaluation of hemolytic activity of *S. aureus* determined spectrophotometrically at 543 nm. **(F)** Alkaline phosphatase activity in *S. aureus* following different treatments. The results indicate a dose-dependent increase in AKP release, demonstrating progressive disruption of bacterial cell wall integrity after treatment, with the combined therapy creating the strongest action. **(G)** β-galactosidase activity in *S. aureus* after treatment with plant extract, CS-AgNPs, and the combined formulation compared to the untreated control. Values are expressed as mean ± SD. Different letters indicate statistically significant differences (*p* < 0.05) using one-way ANOVA with Tukey’s *post hoc* test.

Crystal violet staining showed that the treatment with PE or CS-AgNPs induced only a modest decrease in biomass. However, the most remarkable action detected with the CS-AgNPs/PE treatment was that it nearly completely repressed biofilm establishment (Figure 5A). Similarly, CLSM imaging confirmed partially disturbed structures after plant extract treatment, and a significant loss of biofilm density with CS-AgNPs. Interestingly, CS-AgNPs/PE treatment resulted in near-complete collapse of the biofilm, with only scarce residual aggregates and mostly dead cells (Figure 5B). The quantitative examination further supported these findings. The PE showed a modest reduction (~20–25%), while CS-AgNPs decreased biomass and thickness by approximately half. On the other hand, CS-AgNPs/PE treatment achieved the highest action, reducing biomass and thickness by about 70–80%, significantly increasing roughness, and decreasing cell viability to less than 15% (Figure 5C).

### Effect of CS-AgNPs/PE on the hemolytic potentials in *Staphylococcus aureus*

Hemolysis production is reported to be important for the virulence of *S. aureus* and the major cause of the β-hemolytic phenotype in *S. aureus*. Interestingly, CS-AgNPs/PE treatment completely inhibited hemolytic action, with colonies failing to produce obvious zones of erythrocyte lysis (Figure 5D). A similar pattern was detected in the liquid media, where CS-AgNPs/PE decreased hemolytic capacity of *S. aureus* in a dose-dependent manner (Figure 5E).

### Effect of CS-AgNPs/PE on *Staphylococcus aureus* cell integrity within biofilms

AKP is considered a marker of bacterial cell wall integrity. Our results revealed that when *S. aureus* cells were treated with CS-AgNPs/PE, the concentration of AKP increased significantly, implying its effective inhibitory action on *S. aureus* biofilm (Figure 5F). Similarly, β-galactosidase leakage is indicative of compromised membrane permeability. As displayed in Figure 5G, β-galactosidase activity was significantly greater in the bacterial suspension of CS-AgNPs/PE group.

### Synergistic inhibition of CS-AgNPs and plant extract

The potential synergistic interaction between CS-AgNPs and the plant extract against *S. aureus* was evaluated using the checkerboard assay. Separately, CS-AgNPs and the plant extract exhibited MIC values of 32 μg/mL and 64 μg/mL, respectively. Upon combination, a marked reduction in the MICs of both agents was observed, decreasing to 8 μg/mL for CS-AgNPs and 16 μg/mL for the plant extract. The calculated fractional inhibitory concentration index (FICI) was 0.50, indicating a synergistic interaction between the two agents.

### CS-AgNPs/PE stimulate the *in vitro* osteoblast differentiation of mBMSCs

**Figure 6:**
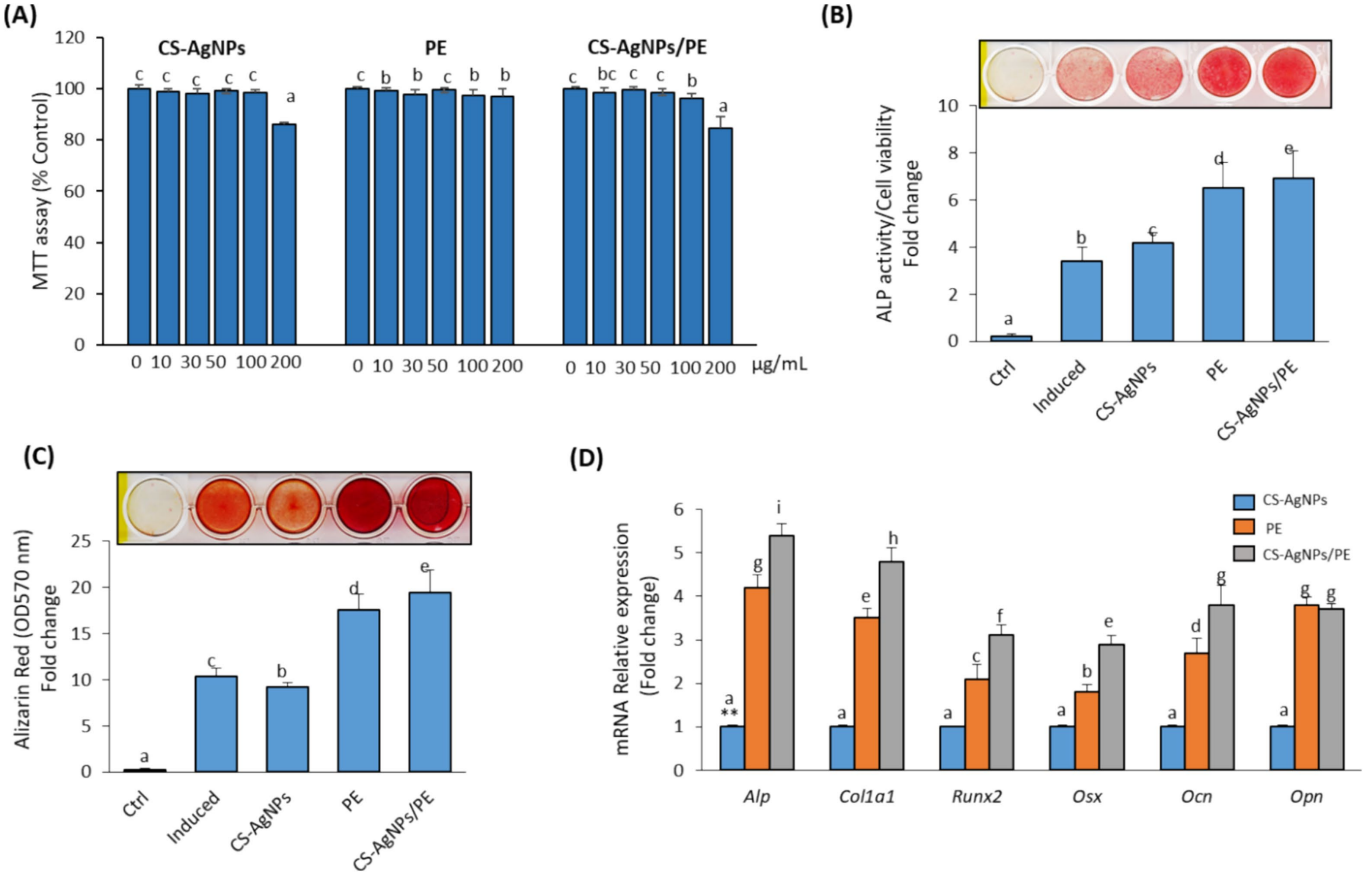
Osteoinductive effect of CS-AgNPs/PE. **(A)** Cytotoxicity of CS-AgNPs, PE, and CS-AgNPs/PE at different concentrations on primary mBMSCs after 48 h of treatment. Cell viability measured by MTT assay. Stimulatory effect of CS-AgNPs, PE, and CS-AgNPs/PE on **(B)** ALP activity and **(C)** matrix mineralization of mBMSCs. Cells were induced to osteoblast differentiation in the absence (Ctrl) or the presence of different treatments (50 μg/mL) for 7 days. ALP and Alizarin red staining images were shown. **(D)** Expression of osteoblast marker genes by qPCR analysis. mBMSCs treated with CS-AgNPs, PE, or CS-AgNPs/PE for 7 days. Gene expression is represented as the fold change relative to induced cells with CS-AgNPs. Values are expressed as mean ± SD. Different letters indicate statistically significant differences (*p* < 0.05) using one-way ANOVA with Tukey’s *post hoc* test. Data are presented as biological replicates (*n* = 6 animals per group), with histomorphometric analysis conducted on three representative tissue sections per group (technical replicates, *n* = 3).

We examined the cytotoxicity of PE, CS-AgNPs, and CS-AgNPs/PE on the cell viability of mBMSCs. No cytotoxicity was observed in animal cells for PE or CS-AgNPs up to 100 μg/mL, whereas significant cytotoxicity was detected only at 200 μg/mL as assessed by MTT assay (Figure 6A). Furthermore, we studied the *in vivo* toxicity of CS-AgNPs. As shown in Supplementary Figure S4A, intraperitoneal injection of either PE, CS-AgNPs, or CS-AgNPs/PE at 100 μg/mL in mice did not show any cytotoxicity after 1 week of injection as assessed by histological examination of liver, kidney, lung, spleen, and heart sections (Supplementary Figure S4).

Interestingly, *in vitro* treatment of mBMSCs with PE alone or in a combination with CS-AgNPs revealed higher stimulatory effect on osteoblast differentiation than CS-AgNPs treatment alone as assessed by measurement of ALP activity (Figure 6B) and Alizarin Red staining for matrix mineralization production (Figure 6C). In consistent, PE alone and CS-AgNPs/PE significantly upregulated the mRNA expression of all examined osteogenic markers by 50 to 80% higher than CS-AgNPs treatment alone (Figure 6D). These data revealed that the osteoinductive effect of CS-AgNPs/PE on mBMSCs attributed to the presence of PE.

### The inhibitory effect of CS-AgNPs/PE on bacterial burden and inflammatory response in the osteomyelitis mouse model

**Figure 7:**
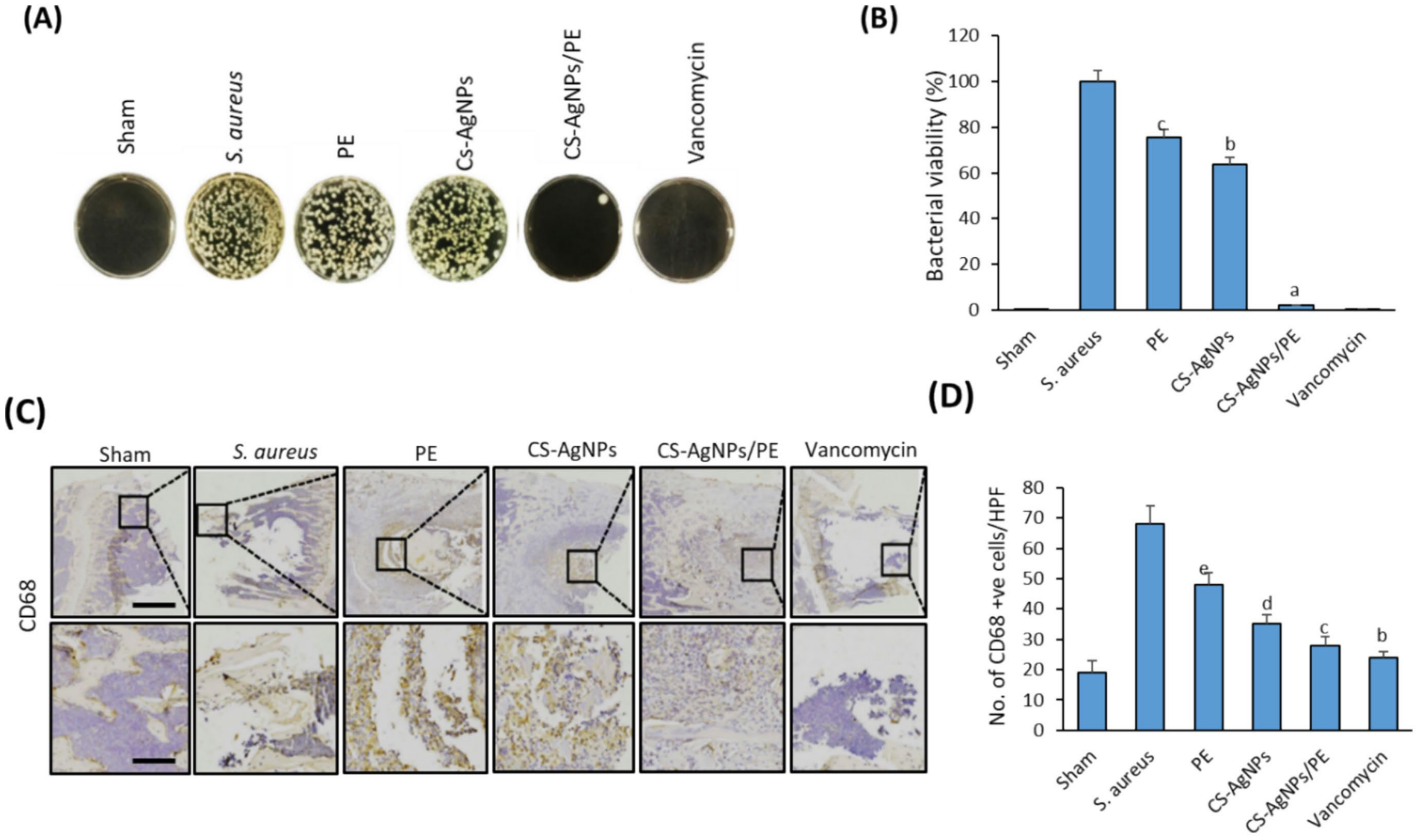
The use of CS-AgNPs/PE for the treatment of osteomyelitis in a mouse model. **(A)** Soybean agar plates presenting the antibacterial effectiveness of different treatments against *S. aureus in vivo* and the matching antibacterial histograms **(B)**. **(C)** CD68 immunohistochemical pictures of bone sections from each treatment group. The scale bar of the left pictures = 250 μm, and the scale bar of right pictures = 50 μm. Values are expressed as mean ± SD. Different letters indicate statistically significant differences (*p* < 0.05) using one-way ANOVA with Tukey’s *post hoc* test. Data are presented as biological replicates (*n* = 6 animals per group), with histomorphometric analysis conducted on three representative tissue sections per group (technical replicates, *n* = 3).

The *in vivo* antibacterial efficiency was evaluated by quantifying bacterial survival within infected bone tissue. As shown in Figure 7A, extensive bacterial growth was observed in the *S. aureus* group, whereas the PE and CS-AgNPs groups showed only modest decreases in bacterial viability, demonstrating that these treatments alone are inadequate to eliminate the infection. On the other hand, CS-AgNPs/PE treatment showed a significant reduction in bacterial burden, with the lowest bacterial survival rate, comparable to that of the vancomycin-treated group (Figures 7A,B).

The inflammatory response in the infected bone niche was evaluated by immunohistochemical staining for CD68, a macrophage-specific marker. Both PE and CS-AgNPs treated groups showed a modest decrease in the number of CD68-positive cells as compared to the infected non-treated group (Figure 7C). Conversely, the CS-AgNPs/PE treated group displayed few CD68-positive macrophages scattered at the periphery of the defect, with minimal indication of inflammatory infiltration and histological appearance closely comparable to the vancomycin treated group (Figure 7D).

### Stimulatory effect of CS-AgNPs/PE on bone regeneration in the IOM mouse model

**Figure 8:**
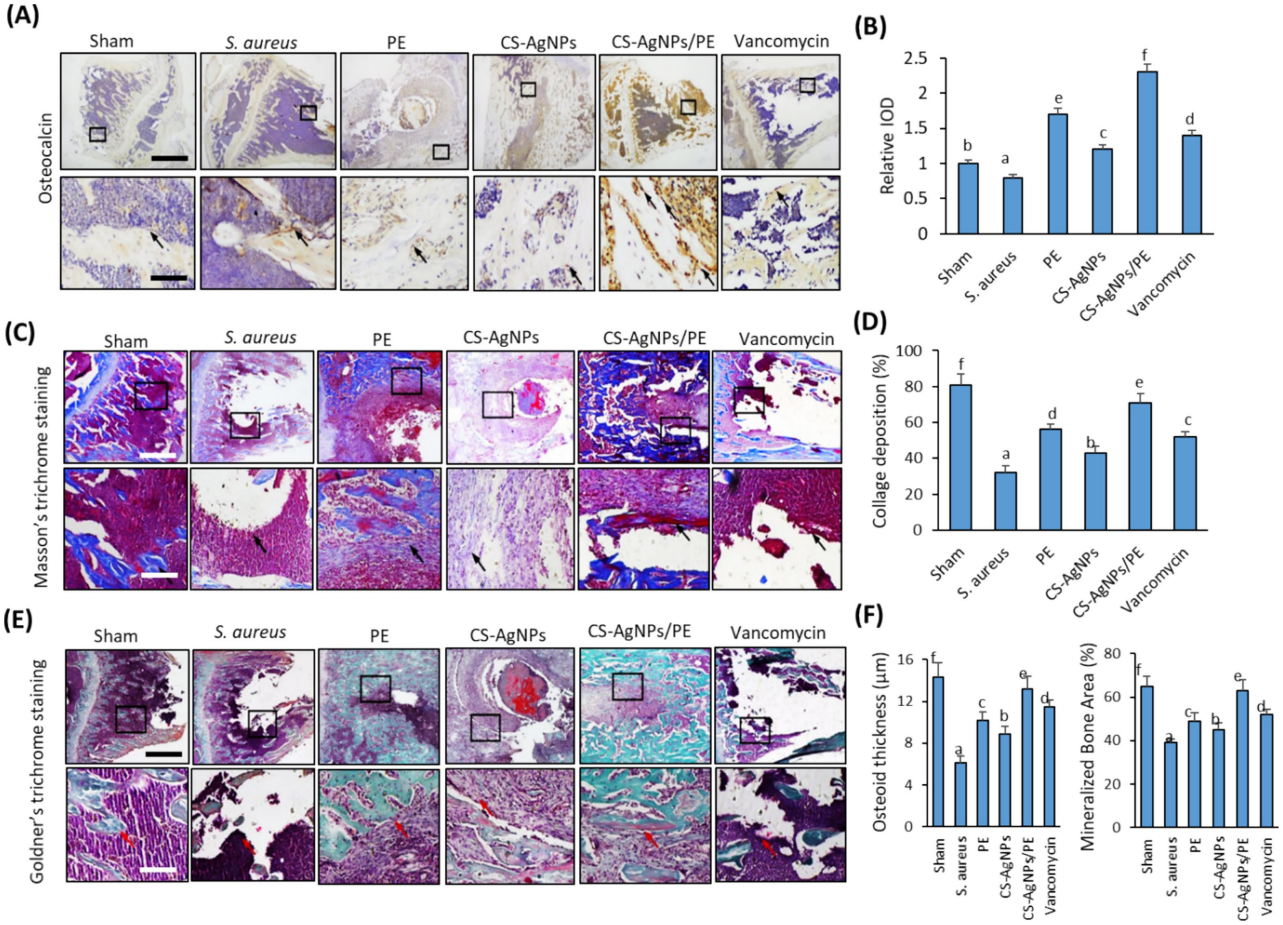
Histopathological and histomorphometric evaluation of osteomyelitis lesions following different treatments. **(A)** Histological examination of infected bone tissues stained with hematoxylin and eosin (H&E), showing inflammatory infiltration and bone destruction in the untreated infected group compared with the treated groups. **(B)** Histological assessment of newly formed bone and inflammatory changes in the treated groups. **(C)** Representative microscopic images demonstrating restoration of bone architecture following treatment. **(D)** Histomorphometric quantification of inflammatory score and bone damage among the experimental groups. **(E)** Goldner’s trichrome staining illustrates osteoid tissue formation and bone regeneration in the different groups. **(F)** Quantitative analysis of newly formed bone area and osteogenic activity in the treated groups. Data are presented as biological replicates (*n* = 6 animals per group), with histomorphometric analysis conducted on three representative tissue sections per group (technical replicates, *n* = 3).

Immunohistochemical staining for osteocalcin (OCN), a late marker of osteoblast maturation and bone matrix deposition, was used to evaluate the osteoinductive effect of CS-AgNPs/PE *in vivo*. As shown in Figure 8A, the infected group displayed severely reduced OCN staining, with only dispersed positive cells noticeable at the defect margins associated with trabecular loss and disturbed bone continuity. Treatment with PE alone showed significantly higher OCN-positive osteoblasts by 45% (dispersed along recently forming trabecular spicules) than CS-AgNPs-treated mice (Figures 8A,B). Remarkably, the CS-AgNPs/PE group confirmed the most robust OCN expression, with strong, continuous staining, well-organized trabecular surfaces, and abundant osteoblasts lining novel bone tissue, as compared to the vancomycin-treated group (Figures 8A,B). Together, these findings designate that CS-AgNPs/PE most efficiently induce osteoblast differentiation and matrix mineralization, resulting in greater bone regeneration in the osteomyelitic environment.

Collagen deposition and extracellular matrix transformation in the defect places evaluated by Masson’s trichrome staining (Figure 8C). Non-treated *S. aureus* infected group showed severe structural disruption, with widespread bone resorption and damage of trabecular continuity associated with irregular blue staining scattered with inflammatory infiltrates (Figure 8C). As shown in Figure 8C, PE group showed better collagen organization, with extra obvious blue staining along nascent trabecular spicules. However, the collagen fibers persisted less mature and less densely packed as compared to healthy bone (Figure 8C). CS-AgNPs treated group revealed incomplete recovery, with small patches of blue-stained collagen associated along irregular bone surfaces as assessed by quantification of collagen deposition (Figure 8C). Interestingly, the CS-AgNPs/PE group revealed the most widespread and mature collagen deposition, with extracellular matrix restoration and actual regeneration of the defect area. Collagen deposition was restored by 80% in the CS-AgNPs/PE group compared to the non-treated control (Figure 8D).

Bone mineralization and maturation were assessed by Goldner’s trichrome staining (Figure 8E). The PE-treated group showed more obvious mineralization, with modest green-stained trabecular regions. Red stained osteoid representing some improvement of matrix maturation, although complete bone quality persisted suboptimal as compared to healthy bone (Figure 8E). CS-AgNPs-treated group presented incomplete development, with dispersed areas of weak green staining along patchy trabeculae, followed by irregular osteoid deposition (Figure 8E). Remarkably, the CS-AgNPs/PE-treated group showed the greatest progressive bone regeneration, with plentiful green staining representing dense, mature mineralized trabeculae spanning the defect site. Red osteoid consistently distributed and the complete bone structure repaired, with well-organized trabecular architecture and minimal sign of fibrous tissue (Figure 8E). Histomorphometric analysis revealed increase in osteoid thickness and mineralized bone in CS-AgNPs/PE treated group by 65 and 72%, respectively over control non-treated group (Figure 8F). Thus, PE alone prompted incomplete mineralization and vancomycin improved osteoid maturation, the CS-AgNPs/PE treated group most efficiently indorses complete bone matrix mineralization and trabecular restoration, closely resembling the histological appearance of normal bone.

## Discussion

The treatment of osteomyelitis is a difficult clinical challenge that requires a treatment strategy with dual antibacterial activity and osteogenic capacity to repair infectious bone defects. In this study, we provided the green formulation CS-AgNPs/PE as a one-step treatment to suppress bacterial infection and enhance bone regeneration in chronic osteomyelitis.

We phytosynthesize CS-AgNPs for the first time using the methanolic plant extract of *C. sinaica*. The pharmacological profile of *C. sinaica* remains underexplored compared with its congeners. The GC–MS analysis showed several major phytoconstituents, including oleic acid, linoleic acid, hexadecanoic acid, and various phenolic and flavonoid derivatives, each of which is known to have distinctive antibacterial actions. For instance, oleic acid, extracted from *Leucas calostachys*, displayed distinctive antibacterial action against *S. aureus* with a MIC of 25.0 mg/mL (28). Similarly, n-hexadecanoic acid isolated from the leaves of *Ipomoea eriocarpa* displayed moderate antibacterial action against *S. aureus* at 50 μg/mL (29). Additionally, 9-octadecanoic acid extracted from Neem oil exhibited powerful antibacterial activity against *S. aureus, E. coli*, *and Salmonella* sp. with MICs from 5 to 20 mg/mL (30).

The phytosynthesized CS-AgNPs were confirmed by a strong peak at 420 nm, which is similar to the documented UV–Visible spectral region of AgNPs between 200 and 800 nm (31). XRD authorized the crystalline nature of CS-AgNPs. The FTIR spectrum of *C. sinaica* extract, showing the incidence of primary biomolecules, such as esters, steroids, terpenes, aliphatic alcohols, fatty acids, and flavonoids, as described by others (32). Additionally, the greater stability of CS-AgNPs established with a zeta potential of −36 mV, which is attributed to the association of anionic stabilizing agents in *C. sinaica* extract with the AgNPs (33). SEM, TEM, and AFM investigations established that CS-AgNPs were spherical in shape with sizes ranging from 22 to 25 nm without any aggregation. These distinctive structures of CS-AgNPs described formerly as optimum properties for phytosynthesized AgNPs with therapeutic prospective (34, 35).

CS-AgNPs/PE established a powerful antibacterial activity against *S. aureus*. The combination treatment expressively decreased both MIC and MBC values compared to either component alone, demonstrating a synergistic interaction between silver ions and phytochemical capping agents. The antibacterial activity of CS-AgNPs against *S. aureus*-induced osteomyelitis could be mediated via diminishing bacterial cell attachment and impairing the integrity of cell membrane structure, resulting in cell death (36, 37). We previously reported that *Farsetia aegyptia*-derived AgNPs significantly inhibit *S. aureus* burden through ROS accumulation, membrane leakage, and disruption of cell-surface structure (16). Furthermore, AgNPs mycosynthesized using *Aspergillus parasiticus* showed potent activity against methicillin-resistant *S. aureus* in both *in vitro* and *in vivo* models (34).

Mechanistically, the combined treatment prompted strong ROS production by 59% as compared with either CS-AgNPs alone 48% or plant extract alone 35%. Furthermore, the obvious elevation in protein and nucleic acid release, higher LDH leakage, and sharp increase in membrane conductivity indicate a complex membrane-disrupting mechanism. Significantly, the CS-AgNPs/PE also decrease hemolytic activity and dramatically increase the release of AKP and β-galactosidase, indicating severe injury of both cell wall and membrane integrity. Therefore, the hybrid nanosystem achieves bactericidal activity via simultaneous ROS production, enzymatic release, and disturbance of bacterial virulence factors (36, 38, 39).

Biofilm development extensively increases bacterial persistence, antibiotic resistance, and infection recurrence. Our data demonstrated that CS-AgNPs/PE inhibit biofilm development and disrupt pre-established biofilm structures of *S. aureus*. Mechanistically, CS-AgNPs/PE combination markedly decreased the hydrophobicity index of *S. aureus* cells to below 20%, a physicochemical modification that likely reduced bacterial adhesion and the initial stages of biofilm development (40). Thus, CS-AgNPs/PE formulation disturbs bacterial communication and adhesion pathways essential for biofilm maturation. In this regard, *Citrus sinensis* peel-mediated AgNPs are reported to disturb *S. aureus* biofilm development and decrease biofilm biomass by ~80% (41).

Several studies reported the anti-inflammatory effect of formulation-containing green nanoparticles, due to the presence of active phytochemicals with anti-inflammatory properties that act as capping agents associated with nanoparticles (16, 17, 42). Consistently, our data showed the anti-inflammatory effect of CS-AgNPs/PE via reducing inflammatory infiltration by macrophages. The anti-inflammatory effect of CS-AgNPs/PE could be attributed to the high level of n-hexadecanoic acid (20%), which is reported to exert a potent anti-inflammatory effect via suppressing cyclooxygenase-1 (COX-1) and cyclooxygenase-2 (COX-2) (43, 44).

We demonstrated the osteoinductive activity of CS-AgNPs/PE via stimulating osteoblast differentiation of BMSCs *in vitro* and enhancing bone regeneration *in vivo*. Interestingly, our *in vitro* and *in vivo* results clearly showed that the osteogenic effect of PE alone was much higher than that of CS-AgNPs alone. Therefore, the osteoinductive effect of CS-AgNPs/PE in bone regeneration might be attributed to the phytochemical components of *C. sinaica* extract. In this context, screening the phytochemicals of PE by GC mass analysis revealed that 70% of the phytochemicals are fatty acids. The potential role of fatty acids in osteoblast formation and bone growth has been investigated in many reports. Fatty acid oxidation increased during osteoblast maturation and is reported to be essential for normal bone acquisition and bone repair (45, 46). Studies have reported that osteoblasts use fatty acids as an energy source, and the skeleton takes up a high fraction of postprandial lipoproteins (47). Impairment of fatty acid catabolism in osteoblasts led to skeletal deficits and affected whole-body fat homeostasis (48). Moreover, the loss function of carnitine palmitoyltransferase 2 (Cpt2) (an obligate enzyme in fatty acid oxidation) in osteoblasts was shown to inhibit osteoblast differentiation and impair postnatal bone acquisition (49).

In addition to fatty acids, *C. sinaica* extract displayed a high level of 5-hydroxymethylfurfural (5-HMF) (8.5%). 5-HMF is characterized by its anti-inflammatory and antioxidant activities (50, 51) and displayed therapeutic effect against inflammatory responses in osteoarthritis chondrocytes (52). Recently, 5-HMF derived from steam-processed *Stauntonia hexaphylla* was shown to induce osteoblastogenesis and to inhibit osteoclastogenesis *in vitro* (53).

Our histomorphometric analysis of bone sections from IOM mice revealed the potential function of CS-AgNPs/PE treatment to increase the *de novo* bone formation, with a strong positive correlation between osteoid thickness and mineralized bone area (*r* ≈ 0.84, *p* < 0.01), where mineralized bone area reached more than 60% of the defect region. Thus, these data provide CS-AgNPs/PE combination as an efficient strategy for improving bone repair under osteomyelitis conditions.

Despite promising results of the current study, several limitations should be acknowledged. Primarily, the present research is a preclinical exploration based on *in vitro* tests and a murine model of osteomyelitis. Consequently, the direct application to humans needs to be proven. The lack of confirmation using human-derived cells or clinical samples might limit the instant clinical application of the outcomes. Future studies, including human tissue models and patient-derived samples, are necessary to authorize the therapeutic potential of the established nano-platform. Additionally, although the treatment demonstrated substantial antibacterial activity, reduced biofilm development, and improved bone-regeneration factors, the results indicate significant therapeutic enhancement, rather than complete suppression of infection. Furthermore, the study design did not comprise a long-term follow-up to assess the stability and continued effects of the treatment. Extended assessment periods in future studies will be necessary to determine the persistence of the therapeutic results and to evaluate possible recurrence of infection. Moreover, it is important to consider that the dosing of vancomycin and CS-AgNPs/PE in the current study was not directly equivalent, which might limit a strict comparative explanation of their therapeutic effectiveness. Vancomycin was administered at a concentration consistent with commonly reported local delivery protocols, whereas the CS-AgNPs/PE n was applied at a lower concentration optimized to achieve biological activity while minimizing potential nanoparticle-associated cytotoxicity. Therefore, the observed outcomes should be interpreted as indicative of therapeutic potential rather than a direct efficacy comparison. Furthermore, the higher MIC value of vancomycin observed in this study exceeds standard susceptibility breakpoints. This might reflect reduced susceptibility of the specific bacterial strain used or variations in experimental conditions. The selected strain was intended to provide a strong infection model; however, this distinction might affect the responsiveness to conventional antibiotics. Therefore, further studies including detailed susceptibility profiling and standardized reference strains are necessary to validate the results. Importantly, our results showed that the osteoinductive actions of CS-AgNPs/PE might be partially attributed to the phytochemical constituents identified in the plant extract, including fatty acids and 5-hydroxymethylfurfural (5-HMF), which have been previously reported to influence osteogenic differentiation and cellular activity. However, it is important to clarify that this interpretation relies on literature-supported associations and the chemical profile obtained in the current study, not direct mechanistic proof. At present, no specific pathway exploration, receptor-level examination, or molecular profiling has been performed to authorize the participation of these compounds in mediating the detected biological effects. Therefore, the suggested mechanism should be considered hypothetical. Further studies are required to explain the precise molecular pathways underlying the osteogenic activity of CS-AgNPs/PE. Furthermore, the applied dosing and the local administration every 48 h for 14 days might not directly translate into a clinically practical approach. This procedure was mainly designed to ensure sustained exposure and to evaluate the therapeutic potential under controlled experimental conditions. Therefore, Future studies are necessary to optimize the delivery strategy, including the development of sustained-release systems and the study of pharmacokinetics to enhance clinical application. Overall, these limitations highlight the necessity for further examinations to extend the current results toward clinical application.

## Conclusion

This study provided a biogenic nanoplatform, CS-AgNPs/PE, that incorporates powerful antibacterial and osteoinductive activities within a single green formulation. The phytochemically capped AgNPs exhibited excellent physicochemical stability, several antibacterial mechanisms, and significant anti-biofilm efficiency against *S. aureus*. Beyond infection management, the formulation induced osteogenic differentiation and enhanced bone regeneration in a mouse model of osteomyelitis, highlighting its dual therapeutic function. The synergistic interaction between silver ions and *C. sinaica* phytochemicals not only improves bactericidal effectiveness but also contributes to anti-inflammatory and pro-osteogenic effects. Together, our results highlight CS-AgNPs/PE as a promising eco-friendly nanotherapeutic medicine for the combined control of chronic osteomyelitis, proposing an inventive bridge between antimicrobial protection and bone tissue regeneration.

## Data Availability

The raw data supporting the conclusions of this article will be made available by the authors, without undue reservation.
