# Supplementary material for: Biogenic Caralluma sinaica-derived silver nanoparticles as a synergistic antibacterial and osteoinductive nanoplatform for osteomyelitis management
Source: Front Med (Lausanne). 2026 May 26;13:1773089. doi: 10.3389/fmed.2026.1773089 (PMC13248622; doi:10.3389/fmed.2026.1773089)
Supplement: Supplementary file 1 [file Data_Sheet_1.PDF]

Supplementary figure S1

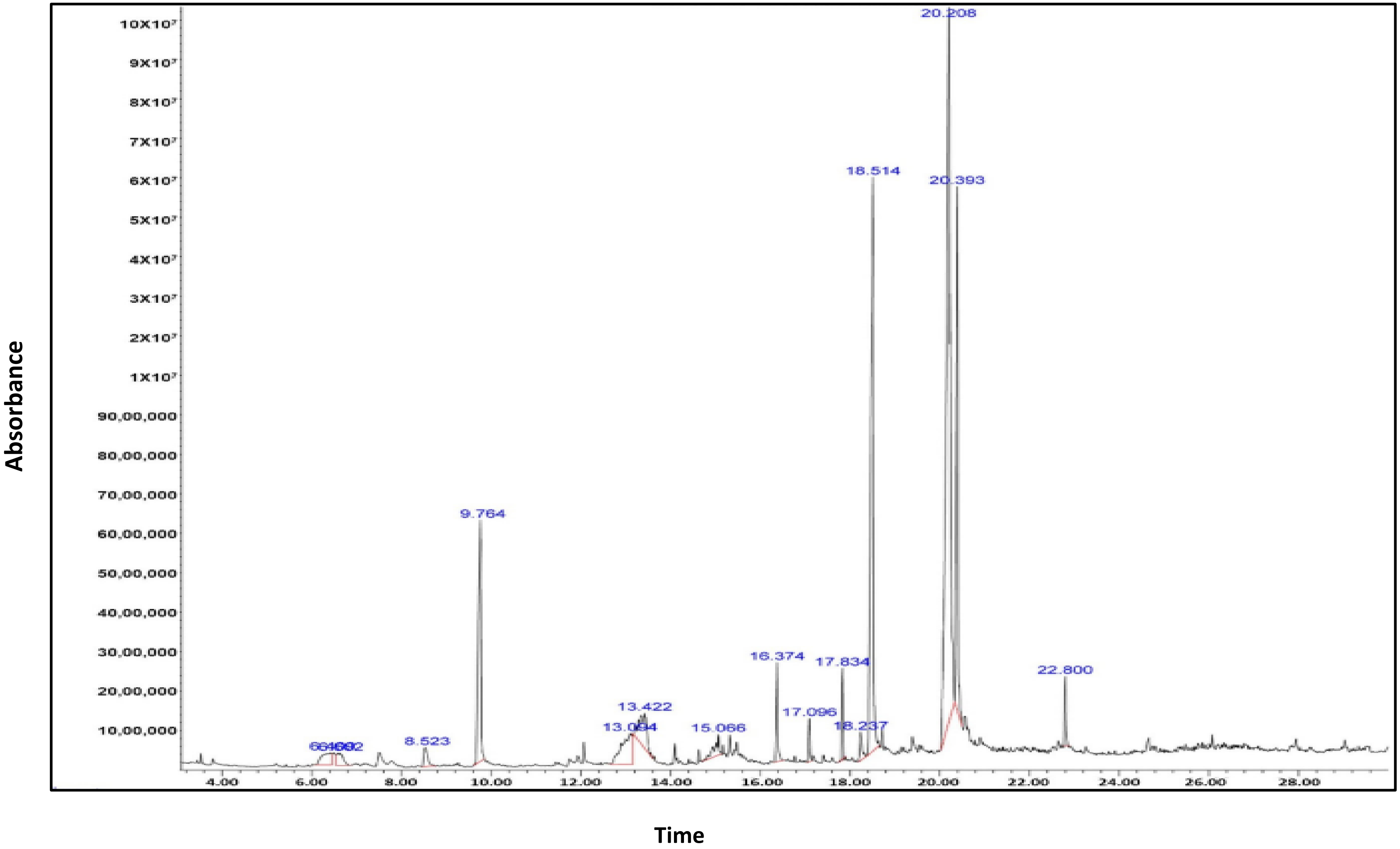

Figure S1

GC–MS chromatogram of aqueous extract of *C. sinaica* .

Supplementary figure S2

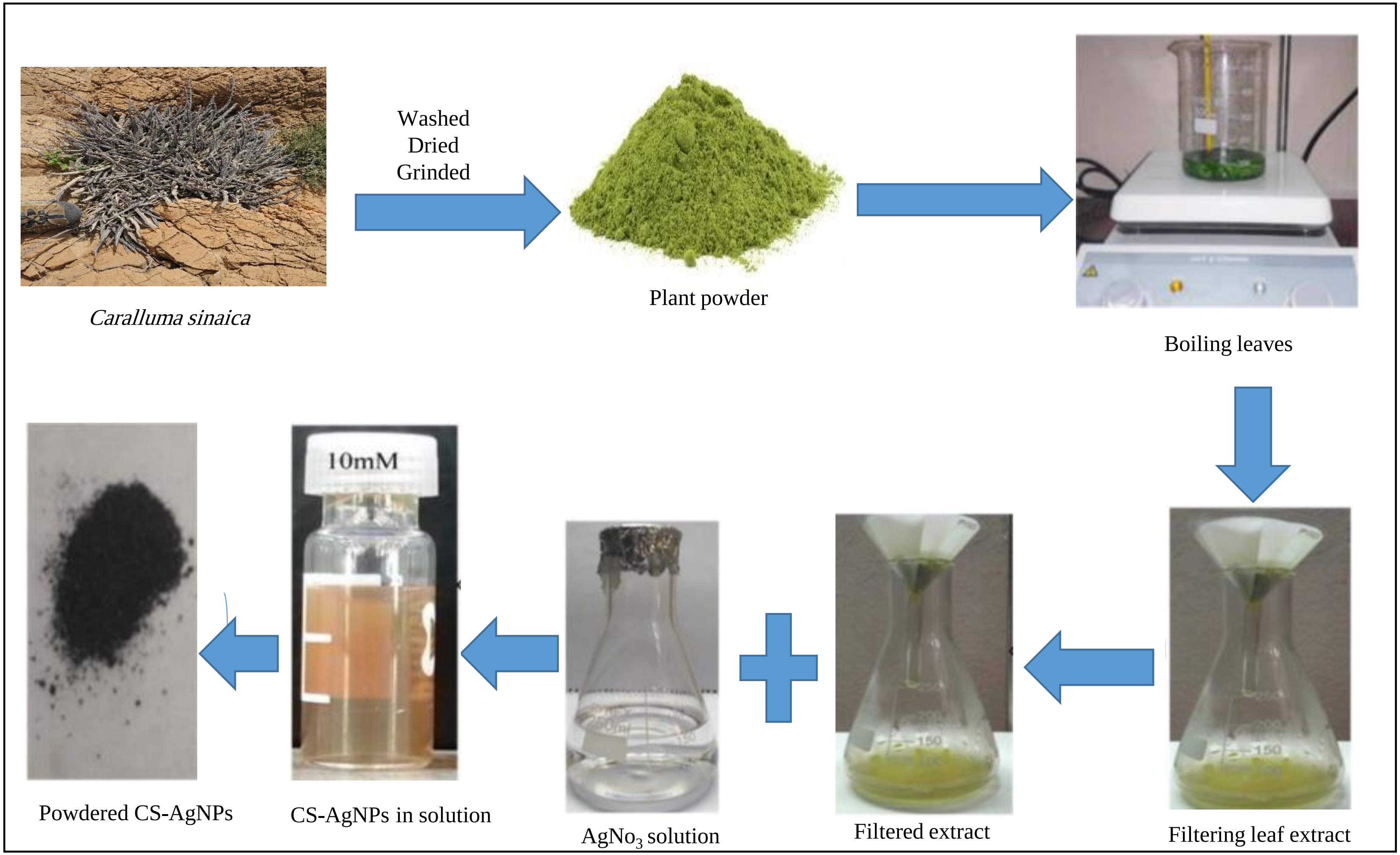

Figure S2. Diagrammatic representation for the phytosynthesis of CS-AgNPs from *C. sinaica* extract.

Supplementary figure S3

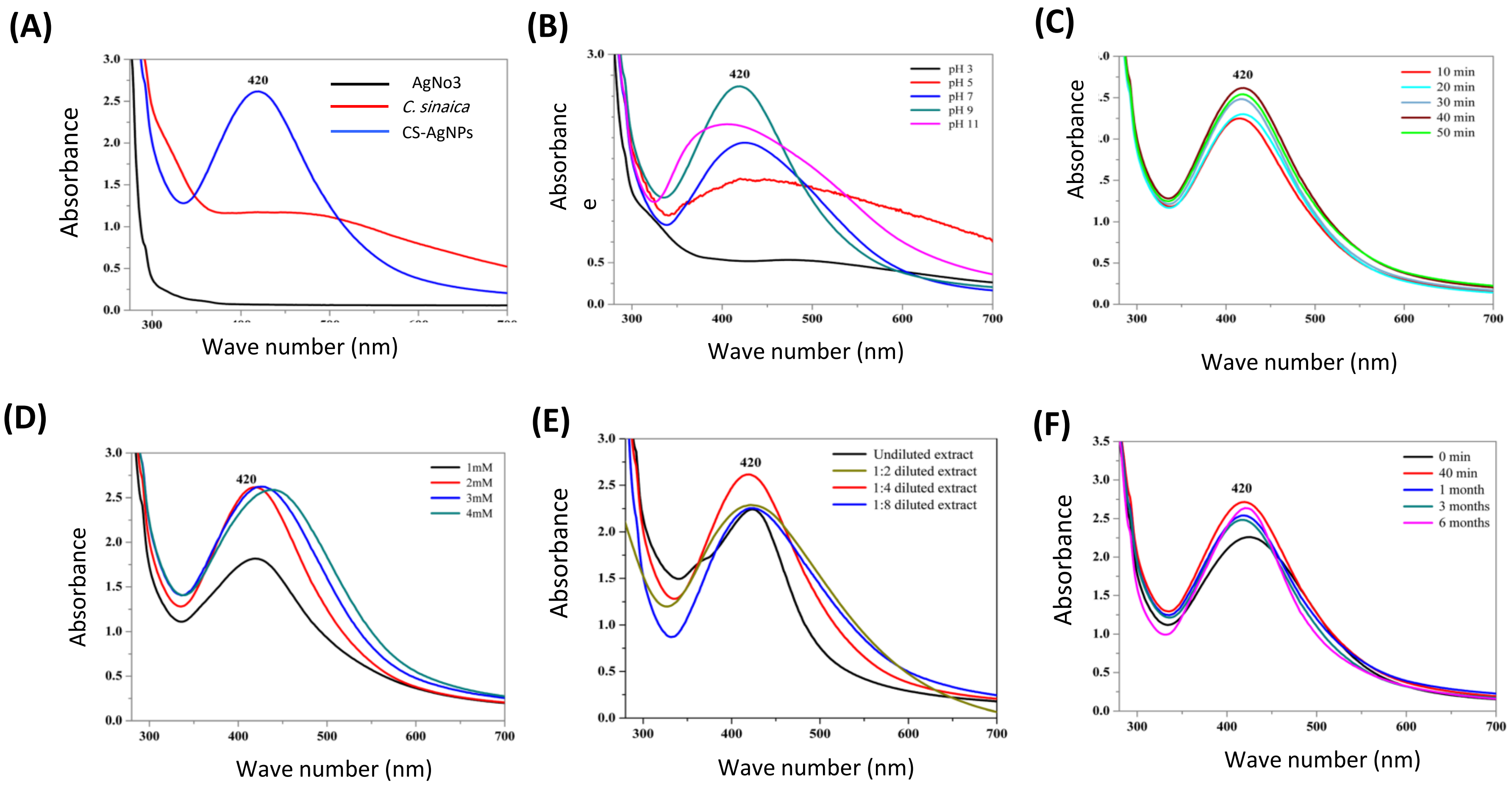

Figure S3. UV–visible spectra of (A) CS-AgNPs phytosynthesized from *C. sinaica* extract at different. (B) pH, (C) exposure time, (D) $AgNO_3$ concentration, (E) extract dilution, and (F) stability.

Supplementary figure S4

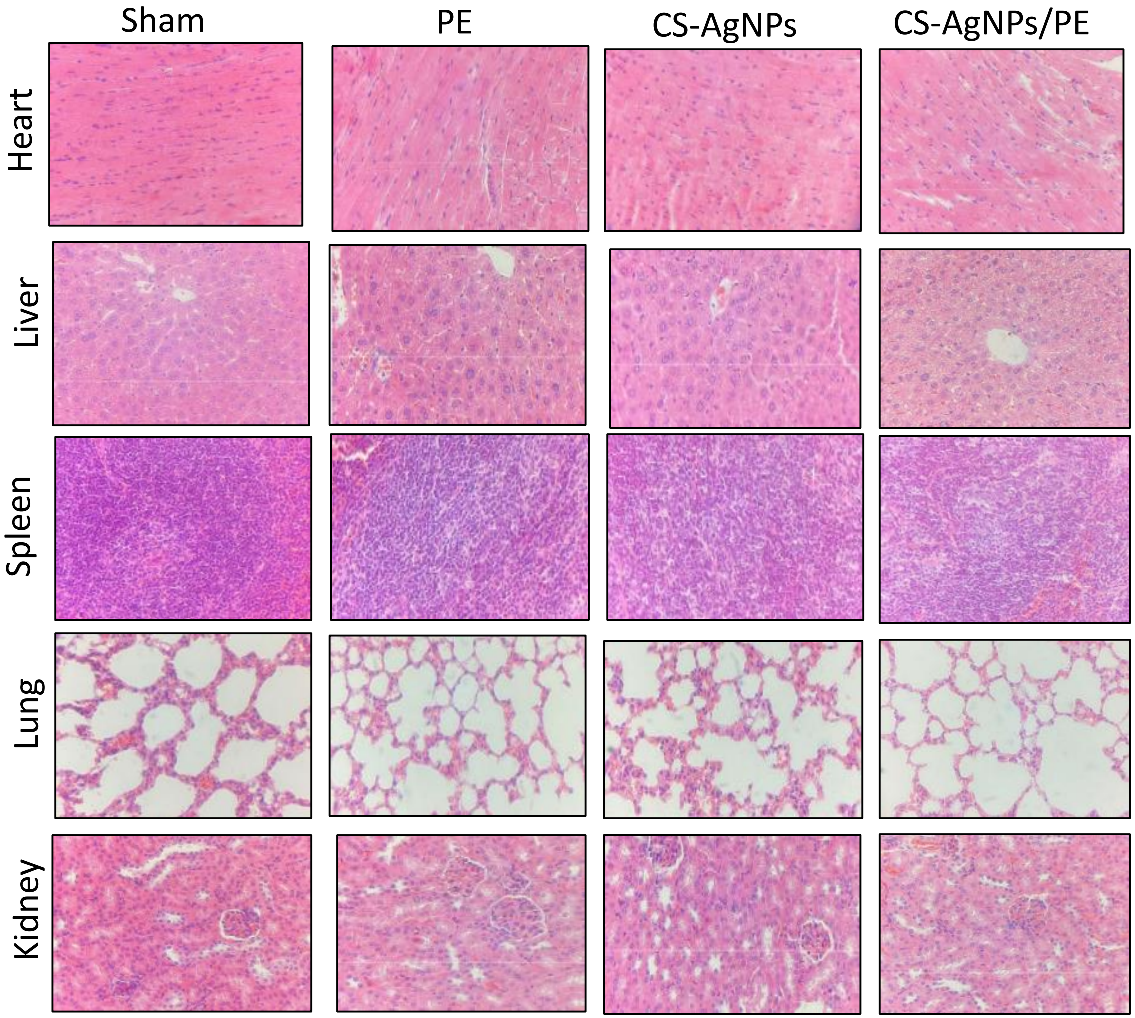

Figure S4. Histology of the heart, liver, spleen, lung, and kidney of mice after different treatments. No noticeable pathological impairment was appeared in the heart, liver, spleen, lung, and kidney of all groups.
