# Supplementary material for: Biogenic Caralluma sinaica-derived silver nanoparticles as a synergistic antibacterial and osteoinductive nanoplatform for osteomyelitis management
Source: Front Med (Lausanne). 2026 May 26;13:1773089. doi: 10.3389/fmed.2026.1773089 (PMC13248622; doi:10.3389/fmed.2026.1773089)
Supplement: Supplementary file 2 [file Data_Sheet_2.pdf]

Supplementary Materials and Methods

Biofilm biomass quantification

Biofilm formation of *S. aureus* evaluated by the crystal violet staining technique as previously described by [1]. Concisely, *S. aureus* (10^7 CFU/ml) treated with different materials in 96-well microplates and incubated at 37°C for 24 h. Biofilms stained with 1% CV for 20 min, washed, and solubilized in 35% acetic acid. The absorbance recorded at 570 nm using a Bio-Rad 680 microplate reader, and biofilm development was determined relative to the control. In addition, the stained biofilms were examined employing light microscope (Olympus IX53, Tokyo, Japan) at 40 x magnification.

Biofilm metabolic activity

Biofilm metabolic activity assessed using the MTT reduction test as described by [2]. After 24 h incubation under the same treatment conditions, wells washed and incubated with 0.5 mg/mL MTT at 37 °C for 2 h. The resulting formazan crystals dissolved in DMSO, and absorbance measured at 570 nm to detect metabolic activity.

Viable cells record in biofilm

The number of viable cells within biofilms was evaluated according [3]. After treatment and incubation for 24 h in 24-well plates, wells washed with PBS, and biofilms were rubbed, homogenized, and serially diluted. Dilutions were cultures on TSA and incubated overnight at 37 °C before counting colonies.

Three-dimensional biofilm visualization and quantification

The 3D structure and extracellular matrix of *S. aureus* biofilms examined by the FilmTracer™ LIVE/DEAD Biofilm Kit. Briefly, Biofilms grown in black 96-well plates under the treatment conditions for 24 h at 37 °C. After removing planktonic cells, biofilms stained with SYTO9 and propidium iodide (3 µL each per 1 mL sterile water) for 20 min in the dark. Then, stained biofilms observed using the ImageXpress ^{micro} high-content screening system. Three-dimensional reconstruction was carried out with Imaris software (v9.0.1), while Image J (v2.14) showed quantitative investigation, comprising biofilm volume, surface roughness, and depth and COMSTAT software [4].

Cell surface hydrophobicity assay

Surface hydrophobicity of *S. aureus* assessed using the microbial adhesion to hydrocarbon (MATH) technique. Briefly, bacterial cultures grown with different

treatments for 24 h, then, washed and adjusted to OD600 ≈ 0.5 in PBS. Suspensions (4mL) mixed with 1mL hexadecane, vortexed for 90 s, and left 20 min. The hydrophobicity rate was measured according to the following equation:

Hydrophobic rate % = (Aa− Ab) /Aa ×100%

Where Aa is the initial absorbance at 600nm, and Ab is the absorption after different treatments [5].

Hemolysis Assay

Fresh erythrocytes were obtained from healthy human blood samples collected in EDTA tubes and washed three times with phosphate-buffered saline (PBS, pH 7.4). A 2% erythrocyte suspension was prepared and incubated with different concentrations of the tested formulations at 37 °C for 1 hour. Phosphate-buffered saline (PBS) was used as the negative control (0% hemolysis), while 1% Triton X-100 was used as the positive control (100% hemolysis). Following incubation, the samples were centrifuged, and the absorbance of the released hemoglobin in the supernatant was measured spectrophotometrically at 540 nm. The percentage of hemolysis was calculated according to the following equation:

Hemolysis (%) = [(A_sample − A_negative control) / (A_positive control − A_negative control)] × 100

Where A_sample represents the absorbance of the tested sample, A_negative control represents the absorbance of PBS-treated erythrocytes, and A_positive control represents the absorbance of erythrocytes treated with 1% Triton X-100 [6] [7].

Release of alkaline phosphatase (AKP) and β-galactosidase

The leakage of intracellular enzymes from *S. aureus* biofilms evaluated according to [8]. Briefly, 100 µL of *S. aureus* cultures (10^7 CFU/ml) in TSB containing 1% sucrose, added to 96-well plates and incubated for 24 h to allow biofilm development. After removing planktonic cells and washing the wells, 200 µL of each treatment was added and incubated for 24 h at 37 °C.The supernatant was collected to analyze AKP and β-galactosidase release by commercial assay kits (Jiancheng, Nanjing, China for AKP; Solarbio, Beijing, China for β-galactosidase). Enzyme leakage levels revealed disturbance of cell integrity within biofilms [9].

SEM of *S. aureus* biofilm

The ultrastructural alterations of *S. aureus* biofilms after different treatments were observed employing FESEM according to [6]. Briefly, sterile glass coverslips placed in 12-well plates, inoculated with bacterial suspensions containing different treatments, and incubated at 37 °C for 24 h. After washing with PBS to remove planktonic cells, the biofilms fixed in 2.5% glutaraldehyde at 4 °C for 5 h, post-fixed in 1% osmium tetroxide, dehydrated through a graded ethanol series, and dried. Samples were mounted, sputter-coated with Au–Pd, and examined using a field-emission scanning electron microscope (S-4800, Hitachi, Tokyo, Japan).

References

1. Fan Q, He Q, Zhang T, Song W, Sheng Q, Yuan Y, Yue T: **Antibiofilm potential of lactobionic acid against Salmonella Typhimurium**. *LWT* 2022, **162**:113461.
2. Jadhav S, Shah R, Bhave M, Palombo E: **Inhibitory activity of yarrow essential oil on Listeria Planktonic cells and biofilms**. *Food Control* 2013, **29**:125-130.
3. Peng F, Hoek EM, Damoiseaux R: **High-content screening for biofilm assays**. *Journal of biomolecular screening* 2010, **15**(7):748-754.
4. Heydorn A, Nielsen AT, Hentzer M, Sternberg C, Givskov M, Ersbøll BK, Molin S: **Quantification of biofilm structures by the novel computer program COMSTAT**. *Microbiology (Reading, England)* 2000, **146 (Pt 10)**:2395-2407.
5. Faleye OS, Sathiyamoorthi E, Lee JH, Lee J: **Inhibitory Effects of Cinnamaldehyde Derivatives on Biofilm Formation and Virulence Factors in Vibrio Species**. *Pharmaceutics* 2021, **13**(12).
6. Lee JH, Kim YG, Lee J: **Inhibition of Staphylococcus aureus Biofilm Formation and Virulence Factor Production by Petroselinic Acid and Other Unsaturated C18 Fatty Acids**. *Microbiology spectrum* 2022, **10**(3):e0133022.
7. Bej AK, Patterson DP, Brasher CW, Vickery MC, Jones DD, Kaysner CA: **Detection of total and hemolysin-producing Vibrio parahaemolyticus in shellfish using multiplex PCR amplification of tl, tdh and trh**. *J Microbiol Methods* 1999, **36**(3):215-225.
8. Zhang W, Margarita GE, Wu D, Yuan W, Yan S, Qi S, Xue X, Wang K, Wu L: **Antibacterial Activity of Chinese Red Propolis against Staphylococcus aureus and MRSA**. *Molecules (Basel, Switzerland)* 2022, **27**(5).
9. Kannappan A, Jothi R, Tian X, Pandian SK, Gowrishankar S, Chunlei S: **Antibacterial activity of 2-hydroxy-4-methoxybenzaldehyde and its possible mechanism against Staphylococcus aureus**. *Journal of applied microbiology* 2023, **134**(7).
