# Supplementary material for: Biogenic Caralluma sinaica-derived silver nanoparticles as a synergistic antibacterial and osteoinductive nanoplatform for osteomyelitis management
Source: Front Med (Lausanne). 2026 May 26;13:1773089. doi: 10.3389/fmed.2026.1773089 (PMC13248622; doi:10.3389/fmed.2026.1773089)
Supplement: Supplementary file 3 [file Data_Sheet_3.PDF]

Supplementary Table 1. MIC of plant extract, CS-AgNPs, and their combination against *S. aureus* induced osteomyelitis and FICI for the CS-AgNPs/ Plant extract combination

Treatment	*S. aureus*			
	MIC alone(µg/ml)	MIC in combination(µg/ml)	FIC	Interaction
Plant extract	64	16	a0.25	
CS-AgNPs	32	8	a0.25	
FICI	-	-	a0.50	Synergitic
